# Peptide Bond Distortions from Planarity: New Insights from Quantum Mechanical Calculations and Peptide/Protein Crystal Structures

**DOI:** 10.1371/journal.pone.0024533

**Published:** 2011-09-16

**Authors:** Roberto Improta, Luigi Vitagliano, Luciana Esposito

**Affiliations:** Istituto di Biostrutture e Bioimmagini, Consiglio Nazionale delle Ricerche (CNR), Napoli, Italy; National Institute for Medical Research, Medical Research Council, United Kingdom

## Abstract

By combining quantum-mechanical analysis and statistical survey of peptide/protein structure databases we here report a thorough investigation of the conformational dependence of the geometry of peptide bond, the basic element of protein structures. Different peptide model systems have been studied by an integrated quantum mechanical approach, employing DFT, MP2 and CCSD(T) calculations, both in aqueous solution and in the gas phase. Also in absence of inter-residue interactions, small distortions from the planarity are more a rule than an exception, and they are mainly determined by the backbone ψ dihedral angle. These indications are fully corroborated by a statistical survey of accurate protein/peptide structures. Orbital analysis shows that orbital interactions between the σ system of C^α^ substituents and the π system of the amide bond are crucial for the modulation of peptide bond distortions. Our study thus indicates that, although long-range inter-molecular interactions can obviously affect the peptide planarity, their influence is statistically averaged. Therefore, the variability of peptide bond geometry in proteins is remarkably reproduced by extremely simplified systems since local factors are the main driving force of these observed trends. The implications of the present findings for protein structure determination, validation and prediction are also discussed.

## Introduction

The structure adopted by a protein is the result of a complex and subtle balance of a number of different stabilization interactions, both intrinsic (i.e. inherent to the polypeptide chain) and environmental (i.e. relative to the interaction with the solvent, ligands and/or other macromolecular partners) [1]. From the chemical-physical point of view, a large variety of different interactions modulate protein structures, such as salt bridges, hydrogen bonds, NH-π interactions, van der Waals interactions, and so on. A full understanding of the factors that determine protein structures would be crucial for many research fields. For instance, it is essential for deciphering protein folding code, for assessing the factors that modulate protein activity, for understanding the effect of mutations on protein structures and, thus, for designing suitable mutants for biotechnological applications [1].

Due to the complexity of the polypeptide chain organization, the structure of protein building blocks is affected by both local and non-local interactions [1]–[3]. Therefore, a preliminary but fundamental step towards a full understanding of the factors determining the protein structural stability is the discrimination between local and non-local effects. In this context, one of the most important and controversial aspects of protein structures regards the peptide bond deviation from planarity [4]–[7], which has also relevant implications for the interpretation of experimental results from many different experimental techniques (NMR, FT Raman, IR, CD) [8]–[12].

**Figure 1:**
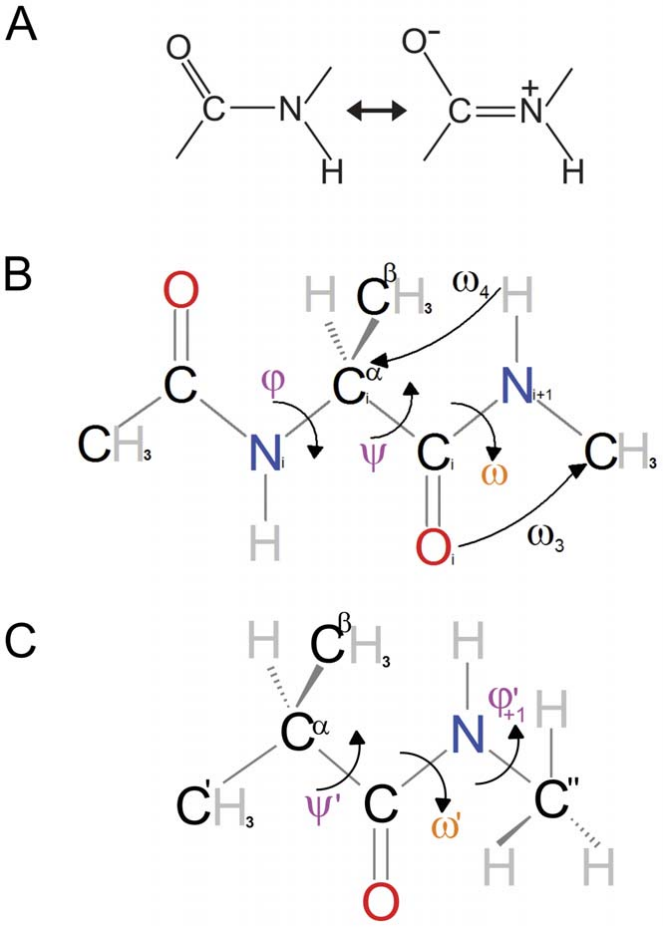
Diagram of model systems. (**A**) Classical representation of the major resonance forms of the peptide bond studied; (**B**) N-acetyl N-methylalaninamide (Ala1); (**C**) Simplified model (Pep). Definitions of backbone conformation angles are shown.

The planarity of the peptide bond represents one of the major constraints imposed on the possible configurations of the polypeptide chain [13]. By postulating that the peptide substituents should lie in the same plane, and, in particular, that ω angle (Fig. 1) could exhibit only perfect cis (0°) or trans (180°) configurations, Pauling & Corey successfully predicted protein secondary structure elements [13]. The peptide bond planarity was traditionally explained by resorting to the resonance model (Fig. 1A), providing the existence of a partial N-C double bond. This simple but powerful picture is the one commonly reported in all biochemistry textbooks, and it is part of the scientific background of most of the researchers in this field.

Actually, already four decades ago, several studies showed that in protein and peptide structures significant distortions of the peptide bond from planarity are allowed [14]–[16]. In more recent years, investigations, carried out by using computational and experimental techniques, have been focused on the entity of peptide bond distortions and on the role that the local context plays in this phenomenon [16]–[22].

In principle, there are different deformation modes of the peptide bond depending on which atom(s) moves out of the plane [14]. Rotations around the C-N bond, that leads to departures of the ω angle from perfect cis (0°) or trans (180°) states, are the most evident ones. More subtle deformations involve pyramidalizations at either the carbonyl carbon (θ_C_) or the nitrogen (θ_N_) atom of the peptide bond. All aspects of peptide deformation have been extensively analyzed but a comprehensive picture of the process is far to be achieved.

Even analyses of peptide bond distortions as function of the local conformation carried out by surveying peptide and protein structure databases have led to different interpretations [16], [21]–[23]. Indeed, MacArthur and Thornton found a correlation between the direction of nonplanar deformation (positive or negative ω values) and the handedness of the protein main-chain twist [16]. On the other hand, taking advantage of the dramatic increase in the number of high- and ultrahigh-resolution protein structures solved in the last decade [24]–[26], we recently observed a correlation between the peptide bond planarity deviation and the dihedral ψ angle [21], [23]. We have also shown that the peptide bond distortions (measured by ω dihedral angle) and carbonyl carbon pyramidalization atom (θ_C_) are related processes that exhibit the same dependency on the ψ angle [23]. Due to the inherent X-ray inability to accurately detect hydrogen atom positions, pyramidalization at the peptide nitrogen atom (θ_N_) cannot be analyzed in protein crystal structures, and remains a rather controversial issue [10], [11], [14], [16], [18], [20], [27].

The electronic and conformational properties of amide bond have been extensively investigated by using computational approaches, often with contrasting results [28], [29]. In fact, studies of the rotational barrier along the C-N bond have either confirmed or challenged the simple resonance model [12], [30]–[32]. Contradictory pictures of charge distributions on the amide group atoms in the planar and twisted forms have also been obtained [12], [31], [33], [34]. Finally, theoretical studies aimed to connect peptide bond deviations with the local conformation have failed in detecting systematic trends [19], [35].

It is thus clear that, notwithstanding all the experimental and computational efforts, a comprehensive picture of the electronic effects modulating the conformational properties of the peptide bond is still lacking and the factors underlying the deviation from a planar geometry are far from being assessed.

Due to the general relevance of the above topics, we have carried out a thorough quantum-mechanical study of different peptide model systems, specifically tailored to allow an easier decomposition of all the chemical-physical effects into play, including solvent effects by means of the Polarizable Continuum Model (PCM) [36]. Different QM methods (DFT, MP2, CCSD(T)) provide a convergent and consistent description of the most relevant stereo-electronic local effects governing the peptide bond planarity and its dependence on other peptide degrees of freedom. In order to dissect the role of local and non-local effects on the geometrical parameters of the peptide bond, we have integrated the theoretical studies with thorough survey of high-resolution protein and peptide structural databases. Both approaches show that small planarity deviations cannot be considered an exception but, on the contrary, occur for most of the states of the Ramachandran space.

## Results

The model system used to study backbone correlations by quantum-mechanics was the peptide-like system denoted as Ala1 (Fig. 1B). Despite being a minimal peptide model, as we shall discuss in the next sections, Ala1 exhibits a significant degree of complexity, since the presence of hydrogen bonding donor (NH) and acceptor (CO) groups favors the formation of intra-residue hydrogen bonds. This may, in turn, affect the geometry of the amide moieties and makes the interpretation of the computational results rather difficult. Therefore, we started analyzing the Pep model, where a methyl group replaced the acetyl moiety at the C-terminus of Ala1 (Fig. 1C).

### Pep: A simple model

#### ω dihedral angle

**Figure 2:**
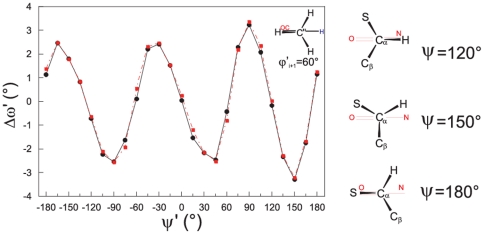
Pep model *in vacuo*: Δω′ variation as a function of ψ′. Results from calculations at the PBE0/6-31G(d) (•) level and MP2/6-31G(d) (▪) level for the conformer ϕ′_i+1_ = 60° (see Fig. S1). On the right side, schematic drawings of conformers of a peptide model characterized by different ψ values are shown. The projections are drawn by looking along the C^α^-C bond (see also Fig. S2). The S substituent stands for the CH_3_-CO-NH- and the CH_3_- group in Ala1 and Pep model, respectively.

We have carried out geometry optimizations of Pep for different values of the C′C^α^CN dihedral angle. Since this dihedral angle corresponds to ψ in Ala1 parent model, we labeled it as ψ′. In particular, we analyzed the ω′ variation as a function of ψ′. We have repeated this analysis for different values of the CNC″H dihedral angle, labeled as ϕ′_i+1_ since this dihedral angle would correspond to ϕ_i+1_ if Pep were inserted in a polypeptide chain. As discussed in detail in the Supporting Information (Text S1), although ϕ′_i+1_ can affect ω′, the qualitative picture does not depend on the orientation of the terminal methyl group and we shall thus discuss here only the results obtained for ϕ′_i+1_ = 60° (Fig. S1). As shown in Fig. 2, according to both PBE0/6-31G(d) and MP2/6-31G(d) calculations ω′ has noticeable deviations from planarity (Δω′ up to 3.5°) and exhibits a - clear sinusoidal dependence on ψ′, with minima and maxima spaced by 60°. The largest Δω′ deviations are found for ψ′ values providing one C^α^-X substituent perpendicular to the peptide plane, whereas structures with one substituent eclipsed to the peptide plane exhibit smaller Δω′ values (Fig. 2 and Fig. S2). Similar trends are displayed by the conformers with ϕ′_i+1_ = 0° or 30° (Fig. S3). Geometry optimizations performed with more extended basis sets (PBE0/6-31+G(d,p), PBE0/6-311+G(2d,2p), MP2/6-31+G(d,p) confirm the PBE0/6-31G(d) and MP2/6-31G(d) results (Fig. S4).

**Figure 3:**
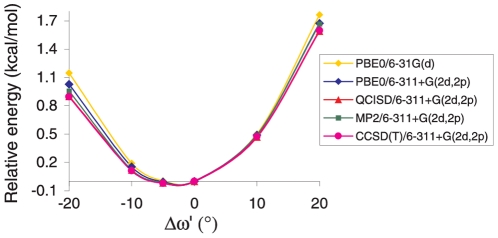
Pep model *in vacuo*: Minimized energy for the ψ′ = 150° conformer at different Δω′ values. Comparison of results from different QM methods.

To further investigate the ψ′/ω′ dependence, we studied in more details the ψ′ = 150° conformer, where a methyl group is perpendicular to the peptide plane and the largest negative value of Δω′ is predicted (Fig. 2). In particular, we performed a series of Single Point energy calculations for different Δω′ values in the range +20°/−20° (Fig. 3). In order to evaluate and isolate the effect of ω′ variations, we kept all the other geometrical degrees of freedom frozen to the values optimized for ψ′ = 120°. This latter conformer, due the symmetrical placement of the methyl groups with respect to the peptide plane (Fig. S2), exhibits a *C_s_* symmetry and a predicted Δω′ = 0°. Furthermore, to definitively check the reliability of PBE0 functional (see also Supporting Information, Text S1) we repeated these calculations by using different Quantum Mechanical methods (PBE0, MP2, QCISD and CCSD(T)). Independently of the computational approach employed, the energy plot presents a minimum for negative values of Δω′ (−2°∼−5°) (Fig. 3). In addition, the curve is significantly asymmetric. For instance, a distortion of Δω′ = +20° from the planarity causes an energy destabilization by ∼0.7 kcal/mol larger than a ω′ distortion of the same entity but in the opposite direction (Δω′ = −20°). It is encouraging that, despite their very limited computational cost, PBE0/6-31G(d) calculations provide a picture close to that of CCSD(T)/6-311+G(2d,2p), also from a quantitative point of view: the energy values predicted by two methods are always within 0.3 kcal/mol (see also Text S1). The results obtained by the different QM methods employed are very similar, as suggesting that the dependence of Δω′ on ψ′ traces back to a very basic mechanism, likely involving the interaction between the electronic orbitals of the peptide group and those of the C^α^ substituents.

Summarizing the results reported in this section, the study of the simple Pep model shows that noticeable deviations from peptide bond planarity are possible, exhibiting a clear sinusoidal dependence on the position of the C^α^-X substituent with respect to the peptide plane (in line with the trend observed in protein structures (ref. 23 and below). From the methodological point of view, simple PBE0/6-31G(d) calculations can provide a reliable estimate of the above phenomena, being in good agreement with the prediction of the other QM methods examined (including DFT calculations with M05-2X functional, Fig. S5), although showing a slightly larger tendency to keep the peptide group planar. As a consequence, we can expect that our subsequent PBE0/6-31G(d) analysis provides a lower bound to the Δω′ values.

#### Carbonyl carbon pyramidalization θ_C_

The distortion from planarity of the peptide linkage, measured by the ω angle, may be also associated to other geometrical parameters. Therefore, we started to analyze peptide parameters directly related to the planarity of the amide moiety such as the carbonyl carbon pyramidalization, which also represents a deviation from peptide planarity. It can be measured by the θ_C_ angle, defined as (ω−ω_3_+π) mod2π where ω_3_ is the dihedral angle defined by the atoms OCN_i+1_C^α^_i+1_ (Fig. 1B) [14].

Previous statistical analysis of ultra-high resolution structures revealed a clear dependence of θ_C_ on the ψ dihedral angle, i.e. the direction of carbonyl carbon atom pyramidalization (above or below the peptide plane) change every 60° of rotation around ψ [4], [21], [23].

**Figure 4:**
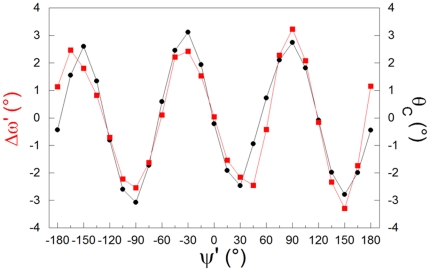
Pep model *in vacuo*: Δω′ (▪) and θ_C_ (•) as a function of ψ′.

Calculations on Pep, carried out at PBE0/6-31G(d) level, confirm the experimental trends (Fig. 4). Fig. 4 also shows that θ_C_ dihedral angle presents the same ψ dependence of Δω. Indeed, a strong positive correlation (R = 0.93) between Δω and θ_C_ is observed with a slope of approximately 1 (y = 0.01+0.93x). This means that the dihedral angle ω_3_ (Fig. 1B) tends to be zero, i.e. the N-CH_3_ (i.e. N_i+1_C^α^_i+1_-like) bond tends to eclipse the C-O bond. However, the correlation between Δω and θ_C_ is significantly larger than that observed in proteins, in line with a general tendency found in the comparison of the experimental data with those derived from calculations on Pep. Despite the similarity of the trends, correlations are enhanced in the simplified system. This suggests that the complexity of protein structures and the interactions with the environment attenuate the entity of these correlations.

### Ala1 in aqueous solution

#### ω dihedral angle

**Figure 5:**
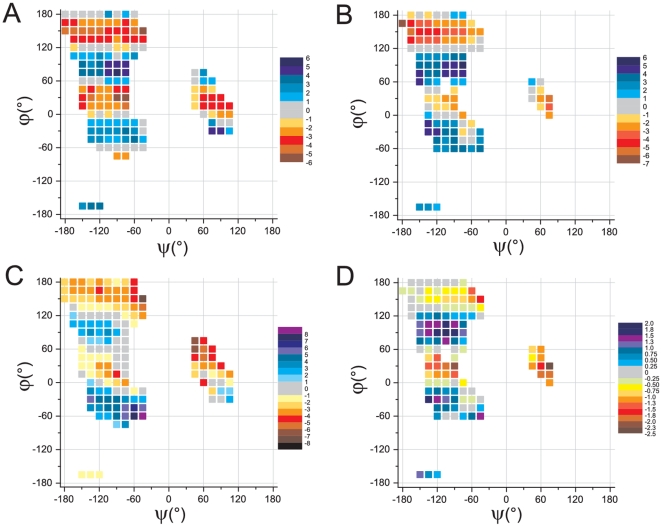
Dependence of Δω and θ_C_ on peptide conformation. Δω *vs.* peptide conformation (**A**) Ala1 model *in solvent* (**B**) high resolution protein structures (resolution better than 1.6 Å; Gly and Pro residues excluded from the database); θ_C_ *vs.* peptide conformation (**C**) Ala1 model *in solvent* (**D**) high resolution protein structures (resolution better than 1.6 Å; Gly and Pro residues excluded from the database). Each experimental point is the average of at least 100 independent values.

After having assessed the reliability of our computational approach, we tackled the study of a peptide system by checking the dependence of Δω on the peptide conformation. To this aim, we fully optimized the geometry of Ala1 in water solution at the PCM/PBE0/6-31G(d) level, on a grid of (15°×15°) in the populated (ϕ,ψ) regions of the Ramachandran plot (Fig. 5A), for which a comparison with the available experimental data is possible. We repeated the surveys of Δω variations in proteins to corroborate our previous analysis [23] by using a much larger structure database (1749 non-redundant protein chains refined at resolution equal or better than 1.6 Å) extracted from the Protein Data Bank [37] (Fig. 5B).

To exclude regions with increased local flexibility, that are less accurate or completely undefined from the structural point of view even in high resolution structures, we omitted residues with higher than average main-chain B-factors (see Materials and Methods for details). A comparative inspection of Figs. 5A–B clearly shows that QM calculations on Ala1 and experimental data provide very similar trends. Both approaches demonstrate that peptide conformations with significant deviations from the planarity (Δω≠0°) are predominant. Indeed, planar peptides are detected only for specific ψ values (∼180°, 120°, 60°, and 0°). Δω exhibits a clear-cut sinusoidal dependence on ψ: positive and negative values of Δω alternate every 60° of ψ. Although some dependency on the ϕ angle is also detectable, the trend is less evident. It is worth noting that in the region with ψ<60°, the values of Δω displayed by conformation with positive and negative ϕ angles are very similar.

In order to better discriminate between intrinsic and environmental effects, we performed our analysis on Ala1 *in vacuo*. As detailed in the Supporting Information (Text S1, Fig. S6), although a sinusoidal dependence of Δω on ψ is found, significant discrepancies between computed and experimental values in protein are observed in the region ϕ<0°, ψ<0° as well as in the regions with ϕ>0° (Fig. S6). The largest discrepancies are found in regions with ψ angles close to 0°. For these conformations the close approach of the NH groups of the two adjacent peptide units (Figs. S2 and S7) allows for the formation of a pseudo hydrogen-bond interaction between the amide moieties. Gas phase calculations likely overestimate the influence of those hydrogen bonds on Ala1 conformation. These interactions are instead reliably described by simple but accurate, continuum solvent models like PCM.

#### Carbonyl carbon pyramidalization θ_C_

Present statistical surveys carried out on high resolution protein structures show, in agreement with previous analyses [21], [23], a clear dependence of θ_C_ on the ψ dihedral angle (Fig. 5D).

As shown in Figs. 5C–D our calculations on Ala1 in aqueous solution provide a picture in good agreement with the experimental one: θ_C_ angle exhibits the same dependence of Δω on ψ, with maximum (and minimum) values appearing every 120°. A positive correlation between Δω and θ_C_ is observed. The linear regression analysis gives a correlation coefficient R = 0.63 (y = −0.0013+0.59x) very close to that provided by the statistics of experimental protein structures [23].

The computational trends obtained for Ala1 in the gas phase are also similar to the experimental ones, also in regions where Δω variations are not in line with the experimental trends (Fig. S8).

### Explaining the experimental and computational trends

In the above paragraphs we have shown that calculations on small model systems and the survey of high resolution protein crystallographic structures provide very similar indications on the dependence of several geometrical parameters on ψ. As discussed in detail in the Supporting Information (Text S1), a survey of small molecule structures from the CSD database [38] (Figs. S9 and S10) provides trends fully consistent with those found in proteins, since Δω and θ_C_ exhibit a clear-cut dependence on ψ.

In order to unveil the basic chemical-physical effects underlying this phenomenon, we have resorted to Natural Bond Orbital (NBO) analysis. NBO theory expresses the first order density matrix in terms of ‘localized’ one-center (core electrons, lone pairs) and two-centers (filled/bonding or empty/antibonding) orbitals, giving a compact and accurate description of the total n-electron density according to simple Lewis-like scheme. Besides being an useful tool for discussing our results in terms of simple ‘Pauling’ resonance structures (see Fig. 1A), the NBO analysis allows also to evaluate how the orientation of the C^α^ substituents affects the properties of the bonding and antibonding orbitals of the amide moiety.

**Figure 6:**
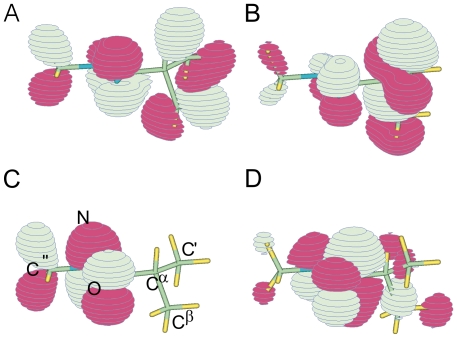
Schematic representation of Pep model molecular orbitals. The three highest energy occupied π orbitals (**A**, **B** mainly corresponding to CO π bonding orbitals, **C** mainly to the Nitrogen Lone Pair) and the lowest energy unoccupied one (**D**, mainly corresponding to CO π* antibonding orbital) are schematically depicted for the ψ′ = 150° conformer with fixed Δω′ = 0°.

A simple inspection of the three highest energy occupied π molecular orbitals (one of them mainly corresponding to the nitrogen lone pair) and the lowest energy unoccupied one, depicted for the ψ′ = 150° conformer in Fig. 6, reveals that the Atomic orbitals of the C^α^ substituents noticeably participate to the amide π system. NBO analysis indicates indeed that the Natural Hybrid Orbitals of the C^α^ substituents (either σ bonding or σ* anti-bonding) interact with the amide π system. Indeed, to get a deeper insight we monitored how the interactions between the Natural Bond Orbitals change as a function of ψ′ in the simplest Pep model, for which all the trends are more easily recognizable. Before analyzing the different degrees of freedom, it is noteworthy that the interaction between the C^α^- moiety (C^α^-X σ and σ* NBOs) and the CO π system (π and π* NBOs) is not negligible with respect to the interaction of the nitrogen lone pair (n NBO) and the CO π system. Indeed, the former interaction is about ∼8–10 kcal/mol (Fig. S11), i.e. the 15% of the nitrogen n→π* amide interaction (∼60–70 kcal/mol, see later in the text).

The survey of the experimental protein structures and QM calculations on Ala1 show that noticeable deviations from the peptide bond planarity are found for well-defined values of ψ.

**Figure 7:**
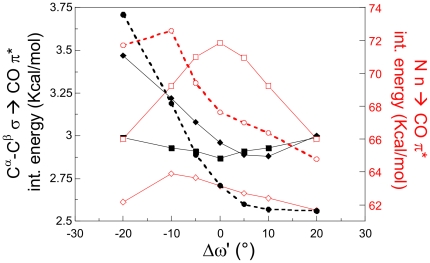
NBO analysis for different Δω′ values. Pep model: Orbital interaction energy for the ψ′ = 120° conformer (squared symbols) and the ψ′ = 150° conformer (diamond symbols) *vs.* Δω′. (▪ ψ′ = 120°, C^α^-C^β^ σ→CO π*; ♦ ψ′ = 150°, C^α^-C^β^ σ→CO π*; □ ψ′ = 120°, N n→CO π*; ◊ ψ′ = 150°, N n→CO π*); Ala1-Solv model: Orbital interaction energy for the (ϕ = −135°, ψ = 150°) conformer *vs.* Δω′. (• C^α^-C^β^ σ→CO π*; ○ N n→CO π*).

NBO analysis indicates indeed that, depending on ψ, the maximum of stability of the amide π system is not reached for a planar arrangement and, on the contrary, small deviations from Δω planarity can be energetically favored. We analyzed the orbital interaction energies for two representative ψ′ conformers of Pep, namely ψ′ = 120° and ψ′ = 150°. Calculations and statistical surveys indicate a preferred Δω = 0° for the former and a maximum (negative) distortion of Δω for the latter. Since our PBE0/6-31G(d)NBO analysis shows that the CO π→C^α^C^β^ σ* interactions provide limited stabilizing effects, we report the most stabilizing interactions involving the CO π system, i.e. the N n→CO π* and the C^α^C^β^ σ→CO π* interactions (Text S1, Fig. 7). For ψ′ = 120° the maximum interaction energy between the nitrogen lone pair n and the CO π* NBO is obtained when the amide moiety is perfectly planar. On the contrary, negative distortions of Δω lead to a significant increase of the N n→CO π* interaction for ψ′ = 150° (Fig. 7). Analogously, C^α^C^β^ σ→CO π* is also favored by negative values of Δω, when ψ′ is 150°.

Although most of our interpretation is based on the Pep model, we have repeated the NBO analysis for two representative structures of Ala1 (ϕ = −135°, ψ = 150° and for ϕ = −60°, ψ = −45°) in order to examine the effect of the substitution of a sp^3^ carbon atom (Pep) with a sp^2^ nitrogen atom (Ala1). We examined two Ala1 conformers which exhibit the largest negative and positive values of Δω. The results indicate that in Ala1 the σ→π* interactions are not significantly weakened (Fig. 7 and Fig. S12). Although the relative weight of the two σ→π* and π→σ* interactions can be different for a specific Cα-X σ bond contribution as well as for specific values of the ϕ angles, the dominance of the σ→π* interactions holds for the whole -CαX_3_ moiety. The trends of the orbital interaction energy vs. Δω obtained for Pep are fully reproduced for Ala1 (Fig. S12), thus confirming that the N n→CO π* interaction is the most significant effect modulating the distortion from the planarity also in this more realistic peptide system.

Collectively, our results indicate that the driving force for the planarity distortion is the maximization of N n→CO π*, and, to a lesser extent, of C^α^-X σ→CO π* interaction energy.

The influence of ψ on the strength of the ‘amide resonance interaction’ can be qualitatively explained by considering the electronic repulsion between the C^α^ substituents and the electrons of the lone pair of the nitrogen atom. This repulsion disturbs the delocalization of the N lone pair in the π system and is expected to be minimal when two C^α^ substituents are in ‘trans’ position with respect to the CN bond and the other one lies in the amide plane without interfering with the π system (ψ = 0°,−120°,120°). In fact, as shown in Fig. S13, the populations of N n and CO π* NBOs exhibit minima and maxima, respectively, for ψ′ = 0°,−120°,120°. On the other hand, the maximum of the repulsion should be observed when two C^α^ substituents are located on the same semi-space containing the CN bond (ψ = 180°,−60°,60°). In these latter conditions, the repulsion may decrease the interaction of the N n orbital with the π system, thus leading to a maximum of the N n and a minimum of the CO π* NBO populations (Fig. S13).

## Discussion

The findings described in previous sections highlight the intricate relationships between different geometrical parameters within the peptide bonds.

We have integrated the results of quantum mechanical calculations of simple peptide model compounds with those of statistical analyses of high-resolution protein crystal structures. Both approaches demonstrate that the orientation of the C^α^ substituents, i.e. the dihedral angle ψ, modulates geometrical parameters, such as Δω and θ_C_ dihedral angles. Furthermore, similar trends can also be detected in the limited but very accurate sample of small molecule crystal structures, thus indicating that the shared overall picture emerging from the three independent analyses (survey on proteins, survey on peptides and computations) is particularly significant.

We have shown that the AOs of the C^α^-H(CH_3_)_2_ group and, to a lesser extent, of the terminal methyl group contribute to the amide π system. Therefore, the geometrical parameters of the amide moiety can also depend on the conformational states of these two flanking groups. It can no longer be taken for granted that the simple rules governing unsubstituted amides still hold for peptide-like systems. It can be thus misleading to focus the attention on the N-C = O moiety only.

The present study shows that, not only are small planarity deviations possible, but they also lead to the stabilization of the peptide bond for most of the states of the Ramachandran space. According to our analysis it is therefore uncorrect to a priori assume that the maximum strength of the ‘amide resonance interaction’ is found for perfectly planar peptide geometries. Depending on ψ, the energetic costs of peptide planarity deviations are no longer symmetric (Figs. 2 and 3). If for a given value of ψ a negative Δω is favored, positive deviations are associated with a destabilization much larger than that expected on the ground of the simple resonance model.

The similarity of the trends of θ_C_/ψ and Δω/ψ indicates the central role of the ψ dihedral angle in modulating peptide planarity as well as the mutual influence among local degrees of freedom.

From the methodological point of view, the results hereby reported also confirm that QM calculations on small peptide models can bring useful insights into the structural properties of large protein structure [39]–[43]. The observed discrepancies between the experimental trends and the theoretical ones resulting from computations in gas phase on the Ala1 model, can be overcome by the inclusion of a continuum solvent model in the calculations. These findings highlight the importance of a correct handling of solute-solvent interactions to reproduce the experimental correlations between peptide bond geometrical parameters.

The main biochemical implication of the present findings is that local effects play a major role in regulating peptide bond flexibility. Even though proteins assume an extremely complicated structure with a hierarchical juxtaposition of basic elements, it is noteworthy that the effects identified in extremely simplified systems (where the side chain is limited to a methyl group) are also detected in statistical analyses of protein and peptide structures containing twenty different aminoacids. Although the conformational properties also depend on the size of the peptide chain [44]–[46] (for example, through the formation of secondary structure elements) or on the side chain type (e.g. through side chain/main chain interactions), we here highlight that very basic electronic effects are nevertheless present and operative, independently of the complexity of the peptide structure examined.

This does not necessarily imply that long-range effects do not play any role in proteins. It is clear that, just to make an example, hydrogen bonds -with other peptide residues or with solvent molecules- involving the Oxygen and the Nitrogen lone pairs, can affect the geometry of a given residue, modulating, inter-alia, Δω or θ_C_. However, as statistical analyses consider a large variety of peptide bonds in different contexts, long-range effects may be averaged out, allowing the emergence of the trends we highlight. Analogously, explicit solute-solvent interactions, whose importance in determining the preferences between different secondary structures has already been highlighted [47], [48], likely affect also peptide bond geometrical parameters. However, most of the residues in protein structures are not exposed to the solvent. Furthermore the impact of explicit water molecules in protein structure surveys is limited due to the averaging of a large number of possible coordination geometries. These considerations explain the agreement found between the prediction obtained by using a Continuum Model, as PCM, and the experimental results. Finally, other short-range non covalent interactions (n→π* interactions) between carbonyl groups of adjacent residues have been suggested to have an effect on protein structure and stability [46], [49]. These orbital interactions could be at play in our Ala1 model and could be involved in determining the slight dependence of planarity deviations from the ϕ angle. The interesting problem of understanding the interplay between these interactions and those we focussed in the present study may be the subject of future investigation.

The elucidation of the role of local effects is important to point out the impact of global structure on specific regions of the polypeptide chain. Indeed, the detection of departures from the trends dictated by local effects may lead to the identification of regions whose properties are strongly influenced by long-range effects. The convergence of long-range effects to endow a specific region of a protein with peculiar properties may be associated with important functional requirements.

The accurate description of the interplay of peptide bond geometrical parameters here achieved also holds implications for protein structure determination, validation and prediction. Indeed, protein structure determinations strongly rely on the inclusion of a priori knowledge of stereochemical parameters [50]. Despite the impressive successes of protein crystallography, the methodological aspects related to the inclusion of these parameters in the refinement are still highly debated [51]–[53]. Our results support the idea that the context dependence of stereochemistry should be introduced in the refinement procedures of protein structures in order to enhance model accuracy [52], [54], [55]. In addition, the correlations here detected may be used as a validation tool for protein structures [26], [56]. In particular, the fine modulation of peptide bond parameters is particularly suited for checking structures determined at high/ultra-high resolution, whose quality assessment may not be assured by standard validation programs that, in the current version, do not consider these aspects. Finally, the conformation-dependent variations of peptide geometry may represent a challenging benchmark for force fields developed for predictive modeling and molecular dynamics investigations. Interestingly, the inclusion of some conformation-dependent geometry [55] significantly improves the accuracy of Rosetta [57], one of the most used and powerful programs in protein structure prediction, as well as the refinement behavior of crystallographic protein structures [58]. These independent results suggest that even the conformation-dependent planarity deviations should be taken into proper account. It is important to note that, although small, the distortion effects can be cumulative, and, therefore, in a polypeptide chain composed of several hundred peptide units, small distortions can lead to substantial structural changes. Just to get a rough idea of the possible significance of these effects, let us consider the crystal structure of a deamidated derivative of ribonuclease A [59] refined at 0.87 Å resolution with a 6.8° standard deviation on the ω angle. By artificially imposing all the trans ω angles to 180°, we obtained a Cα trace that differs from the original one by about 6 Å (calculated on 121 atoms of chain A).

The importance of peptide bonds in biology is not limited to their role in building protein structures. They are also reactive groups, which are substrates of a huge number of proteases. The dependence of the electronic distribution within the peptide bond on the local peptide conformation may be important for understanding the enzyme-substrate recognition process and for the elucidation of the chemical-physical bases of the catalytic process of protein/peptide degradation.

## Materials and Methods

### Systems and strategy

Calculations have been performed by considering peptide models of different complexity. Most of the calculations were conducted on the so-called dipeptide analogue, N-acetyl N′-methylalaninamide (Ala1) (Fig. 1B), which contains all the relevant geometrical parameters to be analyzed.

The conformational space of Ala1 has been sampled by using a grid of (ϕ,ψ) values of (15°×15°). Only (ϕ,ψ) states that are significantly populated in protein/peptide experimental structures were considered. For each state, the geometry of the model has been optimized by keeping ϕ and ψ dihedral angles fixed to their starting values. The minimized structures were used to derive the dependence of the geometrical parameters (ω, θ_C_) on the main chain dihedral angles (ϕ,ψ).

Calculations were also performed on the simplified system Pep (Fig. 1C) in order to get a simpler picture of the influence of the C^α^ substituents on the structural parameters of the amide moiety. As discussed in detail below, Pep has also been used for a preliminary assessment of the accuracy of our computational approach.

### Computational details

The bulk of our computational analysis has been performed at the Density Functional Theory (DFT) level, by using PBE0 hybrid functional [60]. Despite the absence of adjustable parameters, PBE0 provides accurate results for a number of chemico-physical observables in several systems. In particular, PBE0 has shown a remarkable accuracy in the study of polypeptides (see Supporting Information, Text S1, for a more detailed discussion) [39]. Geometries have thus been optimized at the PBE0/6-31G(d) level in the gas phase and at the PCM/PBE0/6-31G(d) level in aqueous solution. The effect of the basis sets have been checked on Pep peptide model by performing geometry optimizations also at the PBE0/6-31+G(d,p) and PBE0/6-311+G(2d,2p) levels.

In order to check the reliability of PBE0 for describing the effects that modulate peptide geometry, we have performed a thorough study of Pep, comparing the PBE0 results with those obtained by other Quantum mechanical methods. We have therefore studied the distortion from planarity of the peptide geometry by using MP2/6-311+G(2d,2p), QCISD/6-311+G(2d,2p), and CCSD(T) 6-311+G(2d,2p) calculations.

We also performed single-point energy calculations by using the recently developed M05-2X functional that is based on simultaneously optimized exchange and correlation functionals, including kinetic energy density. This method has shown very good performance in the treatment of dispersion interactions in noncovalent complexes [61].

Bulk solvent effects on the ground and the excited states have been taken into account by means of the polarizable continuum model (PCM) [36]. In this model the molecule is embedded in a cavity surrounded by an infinite dielectric with the dielectric constant of the solvent (we have used standard dielectric constant 78.39 for water). Geometry optimizations in solution have been performed using UA0 radii for the solute cavity according the UATM model [62]. This approach has already been successfully applied to the study of the conformational properties of oligopeptides in solution.

The computational results have been interpreted with the help of the Natural Bond Orbitals (NBO) analysis [63], [64]. NBO theory expresses the first order density matrix in terms of ‘localized’ one-center (core electrons, lone pairs) and two-centers (filled/bonding or empty/antibonding) orbitals. All calculations have been performed by the Gaussian03 package [65].

### Statistical analyses of the structural databases

Statistical analyses of peptide bond geometrical parameters have been carried out by considering highly accurate structures reported in the Protein Data Bank (PDB) [37]. A preliminary dataset was generated through the PISCES server (http://dunbrack.fccc.edu/pisces) by selecting structures refined at resolution equal or better than 1.6 Å with an R-factor lower than 0.20. This database was built by considering structures sharing sequence identity lower than 25%. Using these criteria, 1749 structures were selected for the 1.6 Å database. Since even very well refined resolution protein structures may contain regions that are locally disordered, peptide bonds were further selected based on their B-factor values. In particular, we excluded either disordered residues (containing atoms with partial occupancy) or residues with an average main-chain B-factor value higher than 1.3 the average B-factor of the main-chain of the entire protein. Only peptide planes in trans conformation were included in the analysis. As Gly and Pro residues have unique chemical structure, they were not considered. The final dataset contained 28791 residues.

Surveys were also conducted on small molecule structures reported in the v5.31 release of the Cambridge Structural Database (CSD) [38]. Data were shown in the Supporting Information section (Text S1).

## Supporting Information

Text S1 **Additional details on: 1. Computations using PBE0 functional; 2. QM studies on peptide models: a survey of literature data and approaches; 3. The influence of ϕ′ on Δω versus ψ; 4. Computations on Ala1 in the gas phase; 5. Additional details on the NBO analysis; 6. Statistics on small molecule crystal structures.**
(DOCX)

Figure S1 **Schematic drawings of conformers of Pep model characterized by ϕ′_i+1_ = 0°,30°,60°.** The projections are drawn by looking along the C″-N bond. The carbonyl group and the hydrogen attached to the peptide nitrogen are shown in red and blue, respectively. It is worth noting that the ϕ′_i+1_ = 90° conformer is equivalent to the ϕ′_i+1_ = 30° conformer in terms of symmetrical arrangement of H substituents with respect to the peptide plane.
(TIF)

Figure S2 **Schematic drawings of conformers of Ala1 model characterized by different ψ values.** The projections are drawn by looking along the C^α^-C bond. The S substituent stands for the CH_3_-CO-NH- group in Ala1 model.
(TIF)

Figure S3 **Pep model** ***in vacuo*** **: Δω′ variation as a function of ψ′.** (**A**) Calculations at the PBE0/6-31G(d) level Results for the three conformers of the terminal C″ methyl group are shown (▴ϕ′_i+1_ = 0°; ▪ϕ′_i+1_ = 30°; •ϕ′_i+1_ = 60°) together with those for the conformer ϕ′_i+1_ = 30° with the OCNH dihedral angle constrained to be 180° (⧫ϕ′_i+1_ = 30° planar form). (**B**) Calculations at the MP2/6-31G(d) level. Results for the three conformers of the terminal C″ methyl group are shown (▴ϕ′_i+1_ = 0°; ▪ϕ′_i+1_ = 30°; •ϕ′_i+1_ = 60°) together with those for the conformer ϕ′_i+1_ = 0° with the OCNH dihedral angle constrained to be 180° (⧫ϕ′_i+1_ = 0° planar form). On the right, schematic drawings of the ϕ′_i+1_ conformers are shown (for a larger version, see Figure S1).
(TIF)

Figure S4 **Pep model** ***in vacuo*** **: Δω′ variation as a function of ψ′.** (**A**) Calculations for the conformer ϕ′_i+1_ = 60° at different levels of theory: •PBE0/6-31G(d), ▪PBE0/6-31+G(d,p) and ⧫PBE0/6-311+G(2d,2p) (**B**) Calculations at the MP2/6-31G(d,p) level for the three conformers of the terminal C″ methyl group: •ϕ′_i+1_ = 60°, ▪ϕ′_i+1_ = 30° and ⧫ϕ′_i+1_ = 0°.
(TIF)

Figure S5 **Pep model** ***in vacuo*** **: Δω′ variation as a function of ψ′.** DFT calculations adopting the M05-2X functional.
(TIF)

Figure S6 **Ala1 model** ***in vacuo*** **.** Dependence of Δω on peptide conformation.
(TIF)

Figure S7 **Dependence of N_i_-N_i+1_ (blue) and N_i_-H_i+1_ (green) on the ψ angle.**
(TIF)

Figure S8 **Ala1 model** ***in vacuo*** **.** Dependence of θ_C_ on peptide conformation.
(TIF)

Figure S9 **CSD small molecule structure survey.** Dependence of peptide bond geometrical parameters on peptide conformation. (**A**) Δω variation as a function of ψ (**B**) θ_C_ variation as a function of ψ.
(TIF)

Figure S10 **CSD small molecule structure survey of tertiary amides (see Table S1).** Dependence of peptide bond geometrical parameters on peptide conformation. (**A**) Δω variation as a function of ψ (**B**) θ_C_ variation as a function of ψ. Only peptide planes in *trans* conformation were included in the analysis. Accurate peptide models were selected by restricting the survey to the structures determined at low temperature (T<200K) with an R-factor lower than 0.05.
(TIF)

Figure S11 **NBO analysis of Pep at the PBE0 level.** (**A**) Orbital interaction energy between the C^α^ σ system and the CO π system as a function of ψ′ (▴ C^α^-C^β^ σ→CO π* + CO π→C^α^-C^β^ σ*; • C^α^-S σ→CO π* + CO π→C^α^-S σ*; ⧫ C^α^-H^α^ σ→CO π* + CO π→C^α^-H^α^ σ*; ▪ Sum of the above three contributions) (**B**) Orbital interaction energy for (▴) C^α^-X σ→CO π* and (•) CO π→C^α^-X σ* as a function of ψ′. With C^α^-X we represent the sum of the three C^α^-C^β^, C^α^-S, and C^α^-H^α^ contributions. It is worth noting that, due to the non-perfect separation between σ and π systems, the C^α^ substituent bonds interact also with the CO σ bond. These small contributions are not included in the figure, explaining why some deviations from the ideal sinusoidal behavior can be found.
(TIF)

Figure S12 **NBO analysis for different Δω′ values in two representative conformers of Ala1-Solv model.** (**A**) Orbital interaction energy *versus* Δω′ for the ϕ = −135°, ψ = 150° conformer (▪ C^α^-H^α^ σ→CO π*; □ CO π→C^α^-H^α^ σ*;♦ C^α^-C^β^ σ→CO π*;◊ CO π→C^α^-C^β^ σ*;○ N n→CO π*); (**B**) Orbital interaction energy *versus* Δω′ for the ϕ = −60°, ψ = −45° conformer (▪ N-C^α^ σ→CO π*;□ CO π→N-C^α^ σ*;♦ C^α^-C^β^ σ→CO π*;◊ CO π→C^α^-C^β^ σ*;○ N n→CO π*).
(TIF)

Figure S13 Population of the CO nonbonding π* orbital (▴) and the N n orbital (▪) as a function of ψ′ in Pep.
(TIF)

Table S1 **Surveys of CSD small molecule crystal structures.**
(DOCX)
