# Correction: Peptide Bond Distortions from Planarity: New Insights from Quantum Mechanical Calculations and Peptide/Protein Crystal Structures

**DOI:** 10.1371/annotation/59ea8bdd-49e0-47a6-a385-d29c9176b70f

**Published:** 2014-01-03

**Authors:** Roberto Improta, Luigi Vitagliano, Luciana Esposito

In Figure 5, on all four panels, the labels on the x axis (psi angle) and the y axis (phi angle) are exchanged. Please see the correct Figure 5 here: http://plosone.org/corrections/pone.0024533.g005.cn.tif

