# Supplementary material for: Peptide Bond Distortions from Planarity: New Insights from Quantum Mechanical Calculations and Peptide/Protein Crystal Structures
Source: PLoS One. 2011 Sep 16;6(9):e24533. doi: 10.1371/journal.pone.0024533 (PMC3174960; doi:10.1371/journal.pone.0024533)
Supplement: Text S1 — Additional details on: 1. Computations using PBE0 functional; 2. QM studies on peptide models: a survey of literature data and approaches; 3. The influence of ϕ′ on Δω versus ψ; 4. Computations on Ala1 in the gas phase; 5. Additional details on the NBO analysis; 6. Statistics on small molecule crystal structures. (DOCX) [file pone.0024533.s001.docx]

**Supporting Information for**

**Peptide bond distortions from planarity: new insights from quantum mechanical calculations and peptide/protein crystal structures**

Roberto Improta*, Luigi Vitagliano, and Luciana Esposito*

Istituto di Biostrutture e Bioimmagini, CNR, via Mezzocannone 16, I-80134 Napoli, Italy

**Contents:**

**Text S1**

**1.** **Computations using PBE0 functional**

**2. QM studies on peptide models: a survey of literature data and approaches**

**3. The influence of ϕ’ on Δω *versus* ψ**

**4. Computations on Ala1 in the gas phase**

**5. Additional details on the NBO analysis**

**6. Statistics on small molecule crystal structures**

**1. Computations using PBE0 functional**

PBE0 is a parameter free hybrid Hartree-Fock /DFT method rooted in the adiabatic connection formula and based on fourth order perturbation theory.

E_XC_^PBE0^= E_XC_^PBE^+1/4(E_X_^HF^-E_X_^PBE^)

E_X_^HF^is the Hartree-Fock exchange and E_X_^PBE^, E_XC_^PBE^ are the exchange and the complete density functional proposed by Perdew, Burke and Ernzerhof (PBE) respectively. The PBE functional is particularly attractive, since it is based on a number of limiting conditions (vide infra) and does not involve empirical parameters. Despite the absence of adjustable parameters, PBE0 provides accurate results for a number of chemico-physical observables in several systems. In particular, PBE0 has shown a remarkable accuracy in the study of polypeptides [1-3]. In fact, it has already been successfully applied to the study of the conformational properties of Gly, Ala, Tyr, and Pro dipeptide analogues, Gly and Ala homopolypeptides, and collagen-like polypeptides containing proline and its derivatives [4-6]. In all of the above systems, the relative energy and the main structural parameters of the different conformers are correctly reproduced. Furthermore, recent studies have shown that it can provide a quite satisfactory description of the subtle balance of non-standard hydrogen bonds and weak dispersive interactions contributing to the stabilization of pyrrolidine dimers [7]. It has also recently shown very good performances in the calculation of the IR spectra of the lowest energy conformer of Alanine and Glycine in the gas phase [8,9]. Actually, the accuracy of different functionals in predicting the relative stability of the most relevant region of the Ramachandran plot of Ala1 has been thoroughly checked in a previous study [10], comparing the DFT results with those provided by MP2, MP4 and, for a simplified system, QCISD calculations. On the balance PBE0 provides the most accurate results with respect to the other functionals examined, PBEPBE, PBELYP, BLYP, B3LYP, MPWPW91, MPW1PW91, HCT407, especially for what concerns the underestimation of the α-helix region with respect to 3_10_-helix and extended structures. The reason of this behaviour has to be sought in the well- known limitations of current density functionals in the treatment of non-covalent interactions. Each single residue in α-helix suffers for a 'local' over-destabilization in DFT calculations. The same problem is found when studying simple formaldehyde dimers at a distance of 3.1 Å, which is even slightly larger than the distance between two consecutive carbonyl carbon atoms in a α structure. The region corresponding to atom/atom distances of ~3-3.5 Å is thus critical and the reliability of any conformational analysis remarkably depends on the accuracy in describing the interactions operative in that region. The better performances of PBE0 with respect to another commonly used hybrid functional as B3LYP depend on the larger accuracy of this former functional in the low density/high-gradient regions that are critical for a correct description of the non-bonded interactions. The Becke exchange functional that, at variance with PBE0, does not obey the Levy condition and the Lieb-Oxford bound exhibits an incorrect asymptotic limit and a quite poor behaviour in that region. The HCTH functional, while exhibiting similar performances to PBE0 for what concerns the difference between C7_eq_ and helix conformers, overestimates the stability of extended structures remarkably. This is probably due to an underestimation of the stability of hydrogen bonds involving amide groups. On the other hand, the PBE0 functional provides a good description of the hydrogen bond strength also in regions distant from the absolute energy minimum, in excellent agreement with MP2 results. As a consequence PBE0, when applied to the study of conformational equilibria in peptides, has always shown a good accuracy. For example, for Tyr PBE0 provide a description of the dependence between main chain and side chain degrees of freedom in good agreement with the experimental indications. This is a comforting indication for what concerns the accuracy of PBE0 in treating non-conventional hydrogen bonds as the N-H/π interactions.

The study of conformational equilibria in n-alkane provides useful insights on the reliability of the different density functionals in treating non-bonding interactions. In this respect a very recent study [11] provides very interesting results, confirming that PBE0 is fairly accurate. When compared with the CCSD(T) theoretical procedure devised by the authors of the above study, the PBE0 error is ~0.25 kcal mol per gauche-interaction (i.e. the energy gap between a trans (t) and a gauche (g) conformer is overestimated by ~0.25 kcal/mol, that between tt and gg conformers by ~0.5 kcal/mol and so on), whereas that of B3LYP larger than 0.3 kcal/mol. Analogously, for all the conformers of n-hexane the RMSD with respect to the best-estimate is ~0.7 kcal/mol at the PBE0 level, ~0.9 kcal/mol at B3LYP level.

On the ground of the considerations reported above it is not surprising that PBE0 provides a very similar picture to CCSD(T) when treating the distortion from the planarity of Pep model. In order to correctly describe this latter process, a theoretical method has to accurately predict the dependence on the local conformation of the amide bond energy and of the interaction between the σ bonds of the C^α^ moiety with the π electrons of the amide. Actually for ψ’=150° only one C^α^-(CH_3_) that perpendicular to the amide plane) significantly interact with the amide π electrons. We have seen above that the error of PBE0 in computing the relative energy of a gauche conformer is only ~ 0.24 kcal/mol. This is the same order of magnitude of the discrepancy between CCSD(T) and PBE0 calculations shown in Figure 3 of the main text.

**2. QM studies on peptide models: a survey of literature data and approaches**

The number of QM mechanical studies devoted to the study of peptides is too large for being exhaustively reviewed here. Schematically we can say that many studies have been devoted to explore the Ramachandran map of a peptide, in order to characterize the different minima and/or to interpret the corresponding experimental spectra (IR, CD, NMR etc), starting from the pioneering studies of Schaefer et al. on Glycine [12-24].

In this field, one important goal was to understand the effect of the side chain on the conformational preferences of the peptide. See, for example, the impressive series of study of Perczel and co-workers [25-28].

Another important topic is instead the study of oligopeptides, starting from dipeptides, in order to verify which are the most relevant minima, concerning especially the on-set of regular secondary structures as α-helix [29-33].

**3. The influence of ϕ’ on Δω versus ψ**

In order to evaluate the influence of the other adjacent dihedral angle, i.e. the CNC''H angle, on the Δω versus ψ correlation, we repeated our analysis for different values of this angle. We labeled it as ϕ'_i+1_ since this dihedral angle would be related to ϕ_i+1_ if Pep is inserted in a polypeptide chain. Three major conformers of the terminal C'' methyl group are possible, differing for the orientation of the hydrogen atoms with respect to the CONH group (Fig. S1). These conformers are characterized by ϕ'_i+1_=0°,30°,60° respectively corresponding to a methyl group with one hydrogen atom eclipsed to the NH bond, perpendicular to the peptide plane, and eclipsed to the CO bond (Fig. S1).

For what concerns the PBE0 results, the only difference among the three ϕ'_i+1_ conformers concerns the absolute value of Δω'. This is always positive for the ϕ'_i+1_=30° conformer, whereas it exhibits both positive and negative values (with an average value of Δω' ~ 0) for the other two conformers. The only significant discrepancies between MP2 and PBE0 concern the ϕ'_i+1_=0° results. In fact, for ϕ'_i+1_=0°, MP2 curve is less regular, and exhibits more marked Δω' maxima. In this conformer, one hydrogen atom of the terminal methyl group is eclipsed with the NH bond and the nitrogen undergoes a noticeable pyramidalization in order to reduce the steric hindrances. [It is worth noting that also other effects can play a role in the staggered/eclipsed equilibrium (see for instance Pophristic and Goodman, 2001). This phenomenon can affect Δω'. Indeed, when the NH bond is forced to lie in the peptide plane (Fig. S3B), the Δω' plot returns to be regular and very similar to that found at the PBE0 level. Analogously, when we applied the same constrain to ϕ'_i+1_=30° conformer (PBE0/6-31G(d) calculations, Fig. S3A), we get a Δω' plot qualitatively similar to that obtained for the other two conformers, i.e. exhibiting both positive and negative values.

For what concerns MO5-2X, the number of studies employing this functional for studying peptides is still limited (see [34], for a challenging case). However, more data are available for other biological systems as nucleobases, for which a good accuracy is found, especially for systems dominated by short-range (< 5Å) dispersion interactions [35,36], which can be considered good test case for local interaction in peptides.

**4. Computations on Ala1 in the gas phase**

We fully optimized the geometry of Ala1 at the PBE0/6-31G(d) level, on a grid of (15°x15°) in the populated (ϕ,ψ) regions of the Ramachandran plot, for which a comparison with the available experimental results is possible. In order to better discriminate between intrinsic and environmental effects, we performed our analysis both in implicit solvent (see the main text) and *in vacuo.* Here we report some additional details on the computations carried out *in vacuo.*

Noticeable and not random deviations of ω dihedral angle from the planarity are recorded. As shown in Fig. S6, they exhibit a clear-cut dependence on the peptide conformation. In the regions with both ϕ<0° and ψ>0°, negative and positive Δω values alternate about every 60° along the ψ axis. Well-recognizable minima and maxima are observed for ψ=60°,120°,180° and for ψ=30°,90°,150°, respectively. This trend is consistent with the systematic variation of Δω with ψ angle previously detected in experimental protein structures [37] and here confirmed (Fig. 5B of the main text). Although some significant differences are present (see below), the general qualitative picture is similar.

The most significant discrepancy between computations on Ala1 in the gas phase and protein crystal structures concerns the dependence of Δω on ϕ. In fact, according to the statistical survey, Δω values are relatively insensitive to ϕ angle changes. A weak dependence is found only in the α-helix region, and it almost disappears when residues belonging to secondary structures elements are excluded from the statistical analysis [37]. On the other hand, according to PBE0 calculations on Ala1, ω deviations from planarity exhibit a larger dependence on ϕ. As a consequence, the values of Δω for 120°< ψ <180°, which are always negative in the experimental reports, are close to zero when ϕ approaches zero. Similarly, as ϕ increases Δω becomes more positive also in the region 60°< ψ<120°.

Large discrepancies between computed and experimental data are also observed in the region with both ϕ<0° and ψ<0° as well as in the regions with ϕ>0°. Indeed, in these latter regions of the plot, calculated Δω values are clearly positive, whereas they are mostly negative in the surveys of experimental protein structures. This finding is likely an additional manifestation of the high dependence of Δω on the ϕ angle detected in the PBE0 minimized structures. In summary, computations on Ala1 in the gas phase confirm that Δω depends on ψ following a sinusoidal oscillation, but the dependence is also modulated by the ϕ angle.

**5. Additional details on the NBO analysis**

In this paragraph we shall discuss NBO results more in detail. Actually, useful hints on the effect of ψ on the electron density of the amide moiety are already provided by the *shape* of the Natural Hybrid orbitals (NHOs) involved in the π NBOs of the CO group. As discussed in the main text, we can conclude that the NHOs of the C^α^ substituents (either σ bonding or σ* anti-bonding) interact with the amide π system.

In order to get a deeper insight into this issue, we examined how the interactions between the σ NBOs of the C^α^H(CH_3_)_2_ moiety and the π NBOs of the Carbonyl group change as a function of ψ' in Pep. We analyzed both the σ→π and π→σ* interactions between a single C^α^-X bond and the CO π system. We also derived an overall picture of the interaction between the whole -C^α^H(CH_3_)_2_ moiety and the CO π system by summing up contributions from C^α^-C and C^α^-H NHO orbital interactions.

Besides the results already commented in the main text, inspection of Figs. S11 and S12 reveals the following findings:

1) Among the σ→π* and π→σ* interactions, the former is more stabilizing. In Fig. S11B, the energy difference and hence the relative weight of the two kind of orbital interactions can be appreciated. The dominance of σ→π* interactions holds for the whole -C^α^H(CH_3_)_2_ moiety as well as for the single C^α^-X bond.

2) For each C^α^-X bond, the interaction energy of its σ system with the CO π system is close to zero when this bond lies in the amide plane (Figs. S2 and S11A).

The largest interaction with the CO π NBOs is exhibited by C^α^-H NBOs, essentially due to the contribution of the C^α^-H σ→ CO π* donation (Fig. S11A). This is indeed larger than that involving C^α^-C σ NBOs due to the minor stability of the C^α^-H σ NBO which makes it closer in energy to the empty CO π* NBO.

Those considerations are true for Pep, where at variance with what found in peptides, no N-C^α^ bond is present. Actually, NBO analysis on Ala1 *(vide infra)* for (φ,ψ)=(-60°,-45°) indicates that the N-C^α^ σ→ CO π* interaction is less stabilizing that CO π→ N-C^α^ σ*, ~1.0 kcal/mol vs. ~1.6 kcal/mol. However, both interactions are less stabilizing than C^α^-H σ→ CO π* and C^α^-CH_3_ σ→ CO π* interactions (Fig. S12). As anticipated in the text, we have repeated our NBO analysis for two representative conformers of Ala1, i.e. those exhibiting the largest negative and positive values of ∆ω. The trends obtained for Pep are fully reproduced (Fig. S12), confirming that the N n→ CO π* interaction is the most significant effect modulating the distortion from the planarity also in this more realistic peptide system.

**6. Statistics on small molecule crystal structures**

We sought an independent corroboration of the dependences of Δω and θ_C_ on peptide conformation by surveying small molecule structures from the Cambridge Structural Database (Fig. S9). To make appropriate comparisons between statistics and calculations, we searched for a fragment similar to the peptide models used in computations.

To select accurate peptide models we restricted the survey to the structures determined at low temperature (T≤ 200K) with an R-factor lower or equal than 0.05. Moreover, powder structures, polymeric structures, disordered structures, and structures with unresolved errors were excluded from the analysis. We considered only peptide planes in *trans* conformation.

Two different searches were carried out on the v5.31 CSD database. In Table S1 the search fragments and the reference codes of the analysed entries are listed. We first searched for a fragment (a) similar to the peptide models used in the calculations, and then we extended our search to a different fragment (b) including tertiary amides.

Although small molecule crystal structures are more accurate than protein structures, they present a larger number of inter-molecular contacts *per* atom. This can influence the conformation and the geometry of the molecules. Nevertheless, the sample analyzed (234 hits from 151 crystal structures; Fig. S9), although limited, clearly exhibits the conformational trend for Δω and θ_C_ already observed in calculations and protein surveys. Indeed, the alternation of positive and negative signs for Δω and θ_C_ along the ψ axis is evident even though there are regions (-120°<ψ<-60° and 60°<ψ<120°) that are low populated in the experimental dataset. Furthermore, negative distortions are predicted for ψ=150° and ψ=30° in agreement with the computed ones. On the contrary, positive distortions are found for ψ=-150° and ψ=-30°, as predicted by our calculations.

Notably, similar results are obtained when the analysis is carried out on a subset of tertiary amides retrieved from the CSD database (Fig. S10). Altogether these findings indicate that the analyzed conformational trends do not depend on the degree of substitution of the amide group.

**References**

1. Barone V, Newton MD, Improta R (2006) Dissociative Electron Transfer in Donor-Peptide-Acceptor Systems: Results for Kinetic Parameters from a Density Functional/Polarizable Continuum Model. J Phys Chem B 110: 12632-12639.

2. Improta R, Antonello S, Formaggio F, Maran F, Rega N, et al. (2005) Understanding Electron Transfer across Negatively-Charged Aib Oligopeptides. J Phys Chem B 109: 1023-1033.

3. Langella E, Improta R, Barone V (2002) Conformational and Spectroscopic Analysis of the Tyrosyl Radical Dipeptide Analogue in the Gas Phase and in Aqueous Solution by a Density Functional/Continuum Solvent Model. J Am Chem Soc 124: 11531-11540.

4. Improta R, Barone V, Kudin KN, Scuseria GE (2001) Structure and Conformational Behavior of Biopolymers by Density Functional Calculations Employing Periodic Boundary Conditions. I. The Case of Polyglycine, Polyalanine, and Poly-a-aminoisobutyric Acid in Vacuo. J Am Chem Soc 123: 3311-3322.

5. Improta R, Mele F, Crescenzi O, Benzi C, Barone V (2002) Understanding the role of stereoelectronic effects in determining collagen stability. 2. A quantum mechanical/molecular mechanical study of (proline-proline-glycine)n polypeptides. J Am Chem Soc 124: 7857-7865.

6. Benzi C, Improta R, Scalmani G, Barone V (2002) Quantum mechanical study of the conformational behavior of proline and 4R-hydroxyproline dipeptide analogues in vacuum and in aqueous solution. J Comput Chem 23: 341-350.

7. Improta R, Berisio R, Vitagliano L (2008) Contribution of dipole-dipole interactions to the stability of the collagen triple helix. Protein Sci 17: 955-961.

8. Balabin RM Conformational Equilibrium in Glycine: Experimental Jet-Cooled Raman Spectrum. J Phys Chem Lett 1: 20-23.

9. Balabin RM The identification of the two missing conformers of gas-phase alanine: a jet-cooled Raman spectroscopy study. Phys Chem Chem Phys 12: 5980-5982.

10. Improta R, Barone V (2004) Assessing the reliability of density functional methods in the conformational study of polypeptides: The treatment of intraresidue nonbonding interactions. J Comput Chem 25: 1333-1341.

11. Gruzman D, Karton A, Martin JML (2009) Performance of Ab Initio and Density Functional Methods for Conformational Equilibria of CnH2n+2 Alkane Isomers (n = 4-8). J Phys Chem A 113: 11974-11983.

12. Sellers HL, Schafer L (1978) Investigations concerning the apparent contradiction between the microwave structure and the ab initio calculations of glycine. J Am Chem Soc 100: 7728-7729.

13. Schaefer L, Sellers HL, Lovas FJ, Suenram RD (1980) Theory versus experiment: the case of glycine. J Am Chem Soc 102: 6566-6568.

14. Schaefer L, Van Alsenoy C, Scarsdale JN (1982) Ab initio studies of structural features not easily amenable to experiment. 23. Molecular structures and conformational analysis of the dipeptide N-acetyl-N'-methyl glycyl amide and the significance of local geometries for peptide structures. J Chem Phys 76: 1439-1444.

15. Klimkowski VJ, Schaefer L, Momany FA, Van Alsenoy C (1985) Ab initio studies of structural features not easily amenable to experiment. 44. Local geometry maps and conformational transitions between low-energy conformers of N-acetyl-N'-methylglycinamide. An ab initio study at the 4-21G level with gradient relaxed geometries. THEOCHEM 25: 143-153.

16. Scarsdale JN, Van Alsenoy C, Klimkowski VJ, Schaefer L, Momany FA (1983) Ab initio studies of molecular geometries. 27. Optimized molecular structures and conformational analysis of Nalpha -acetyl-N-methylalaninamide and comparison with peptide crystal data and empirical calculations. J Am Chem Soc 105: 3438-3445.

17. Schaefer L, Klimkowski VJ, Momany FA, Chuman H, Van Alsenoy C (1984) Conformational transitions and geometry differences between low-energy conformers of N-acetyl-N'-methyl alaninamide: an ab initio study at the 4-21G level with gradient relaxed geometries. Biopolymers 23: 2335-2347.

18. Head-Gordon T, Head-Gordon M, Frisch MJ, Brooks C, III, Pople J (1989) A theoretical study of alanine dipeptide and analogs. Int J Quantum Chem, Quantum Biol Symp 16: 311-322.

19. Brooks C, III, Case DA (1993) Simulations of peptide conformational dynamics and thermodynamics. Chem Rev (Washington, D C) 93: 2487-2502.

20. Head-Gordon T, Head-Gordon M, Frisch MJ, Brooks CL, III, Pople JA (1991) Theoretical study of blocked glycine and alanine peptide analogs. JAm Chem Soc 113: 5989-5997.

21. Jalkanen KJ, Suhai S (1996) N-Acetyl-L-alanine N'-methylamide: a density functional analysis of the vibrational absorption and vibrational circular dichroism spectra. Chem Phys 208: 81-116.

22. Kukushkin AK, Jalkanen KJ (2010) Role of quantum chemical calculations in molecular biophysics with a historical perspective. Theor Chem Acc 125: 121-144.

23. March NH, Matthai CC (2010) The application of quantum chemistry and condensed matter theory in studying amino-acids, protein folding and anticancer drug technology. Theor Chem Acc 125: 193-201.

24. Gorbunov Roman D, Nguyen Phuong H, Kobus M, Stock G (2007) Quantum-classical description of the amide I vibrational spectrum of trialanine. J Chem Phys 126: 054509.

25. Perczel A, Farkas O, Csizmadia IG (1996) Peptide Models. 18. Hydroxymethyl Side-Chain Induced Backbone Conformational Shifts of L-Serine Amide. All ab Initio Conformers of For-L-Ser-NH2. J Am Chem Soc 118: 7809-7817.

26. Viviani W, Rivail JL, Perczel A, Csizmadia IG (1993) Peptide models. 3. Conformational potential energy hypersurface of formyl-L-valinamide. J Am Chem Soc 115: 8321-8329.

27. Kang YK (2006) Conformational Preferences of Non-Prolyl and Prolyl Residues. J Phys Chem B 110: 21338-21348.

28. Ebata T, Hashimoto T, Ito T, Inokuchi Y, Altunsu F, et al. (2006) Hydration profiles of aromatic amino acids: conformations and vibrations of L-phenylalanine-(H2O)n clusters. Phys Chem Chem Phys 8: 4783-4791.

29. Perczel A, Csizmadia IG (1995) Searching for the simplest structural units to describe the three-dimensional structure of proteins. Int Rev Phys Chem 14: 127-168.

30. McAllister MA, Perczel A, Csaszar P, Csizmadia IG (1993) Peptide models 5. Topological features of molecular mechanics and ab initio 4D-Ramachandran maps. Conformational data for Ac-L-Ala-L-Ala-NHMe and For-L-Ala-L-Ala-NH2. THEOCHEM 107: 181-198.

31. Cheung M, McGovern ME, Jin T, Zhao D-C, McAllister MA, et al. (1994) Peptide Models 10. Topological features of molecular mechanics and ab initio 6D-Ramachandran maps. Conformational data for Ac-L-Ala-L-Ala-L-Ala-NHMe and For-L-Ala-L-Ala-L-Ala-NH2. THEOCHEM 115: 151-224.

32. Pohl G, Beke T, Borbely J, Perczel A (2006) Prediction of Folding Preference of 10 kDa Silk-like Proteins Using a Lego Approach and ab Initio Calculations. J Am Chem Soc 128: 14548-14559.

33. Topol IA, Burt SK, Deretey E, Tang T-H, Perczel A, et al. (2001) alpha - and 310-Helix Interconversion: A Quantum-Chemical Study on Polyalanine Systems in the Gas Phase and in Aqueous Solvent. J Am Chem Soc 123: 6054-6060.

34. van Mourik T (2008) Assessment of Density Functionals for Intramolecular Dispersion-Rich Interactions. J Chem Theory Comput 4: 1610-1619.

35. Hohenstein EG, Chill ST, Sherrill CD (2008) Assessment of the Performance of the M05-2X and M06-2X Exchange-Correlation Functionals for Noncovalent Interactions in Biomolecules. J Chem Theory Comput 4: 1996-2000.

36. Zhao Y, Schultz NE, Truhlar DG (2006) Design of Density Functionals by Combining the Method of Constraint Satisfaction with Parametrization for Thermochemistry, Thermochemical Kinetics, and Noncovalent Interactions. J Chem Theory Comput 2: 364-382.

37. Esposito L, De Simone A, Zagari A, Vitagliano L (2005) Correlation between omega and psi dihedral angles in protein structures. J Mol Biol 347: 483-487.
