# Supplementary figures and images for: Peptide Bond Distortions from Planarity: New Insights from Quantum Mechanical Calculations and Peptide/Protein Crystal Structures
Source: PLoS One. 2011 Sep 16;6(9):e24533. doi: 10.1371/journal.pone.0024533 (PMC3174960; doi:10.1371/journal.pone.0024533)

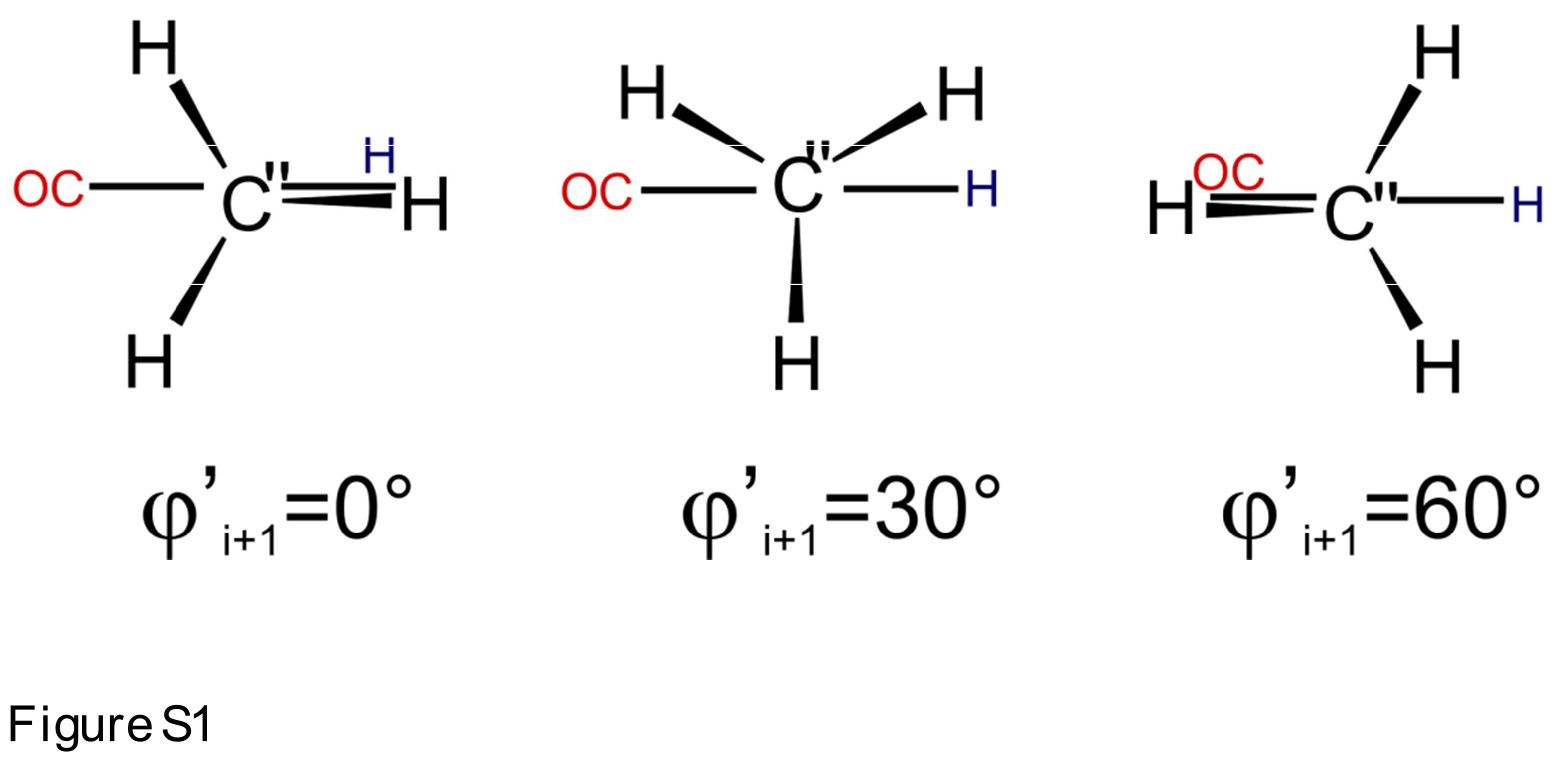

Supplement: Figure S1 — Schematic drawings of conformers of Pep model characterized by ϕ′i+1 = 0°,30°,60°. The projections are drawn by looking along the C″-N bond. The carbonyl group and the hydrogen attached to the peptide nitrogen are shown in red and blue, respectively. It is worth noting that the ϕ′i+1 = 90° conformer is equivalent to the ϕ′i+1 = 30° conformer in terms of symmetrical arrangement of H substituents with respect to the peptide plane. (TIF) [file pone.0024533.s002.tif]

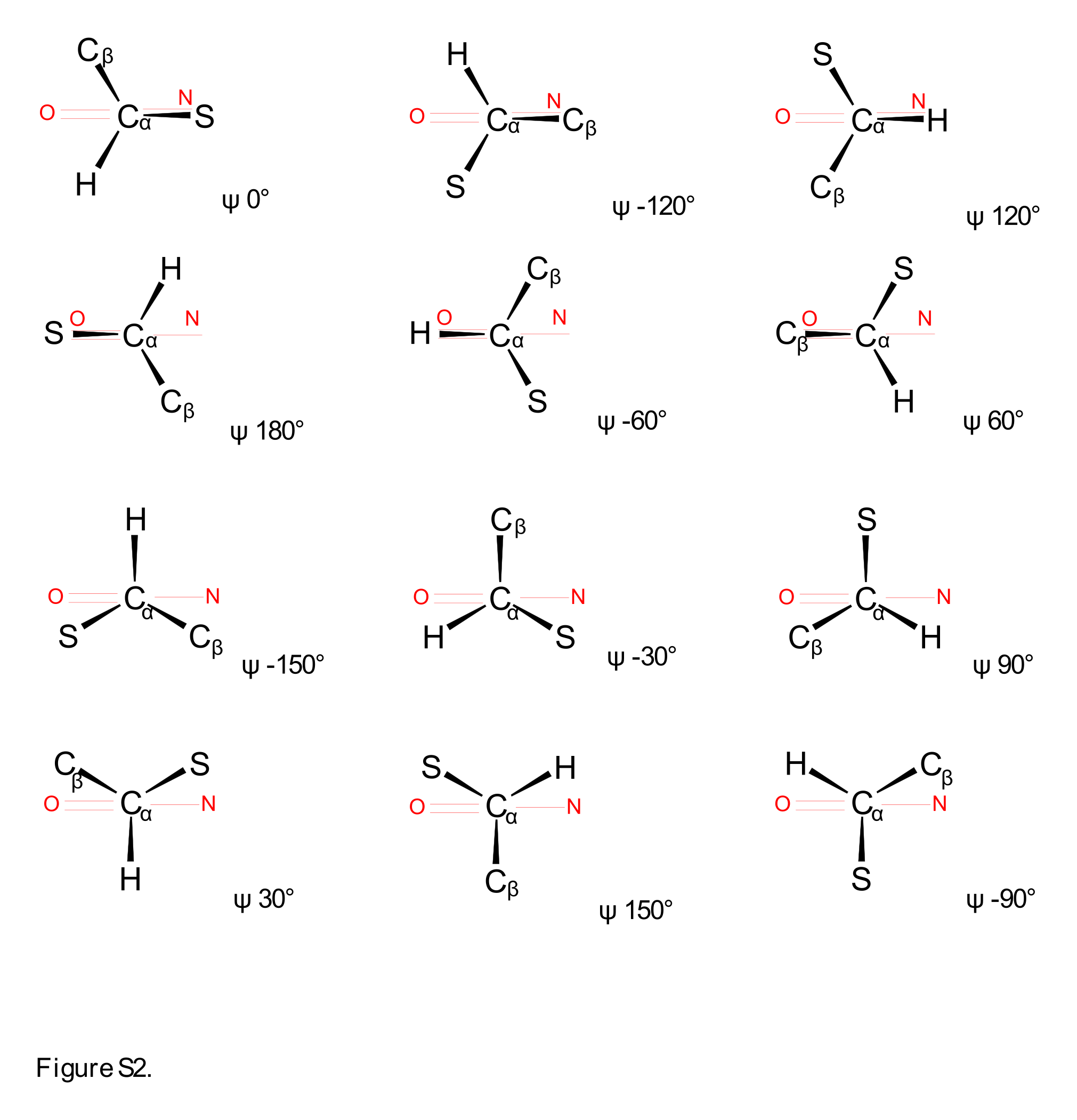

Supplement: Figure S2 — Schematic drawings of conformers of Ala1 model characterized by different ψ values. The projections are drawn by looking along the Cα-C bond. The S substituent stands for the CH3-CO-NH- group in Ala1 model. (TIF) [file pone.0024533.s003.tif]

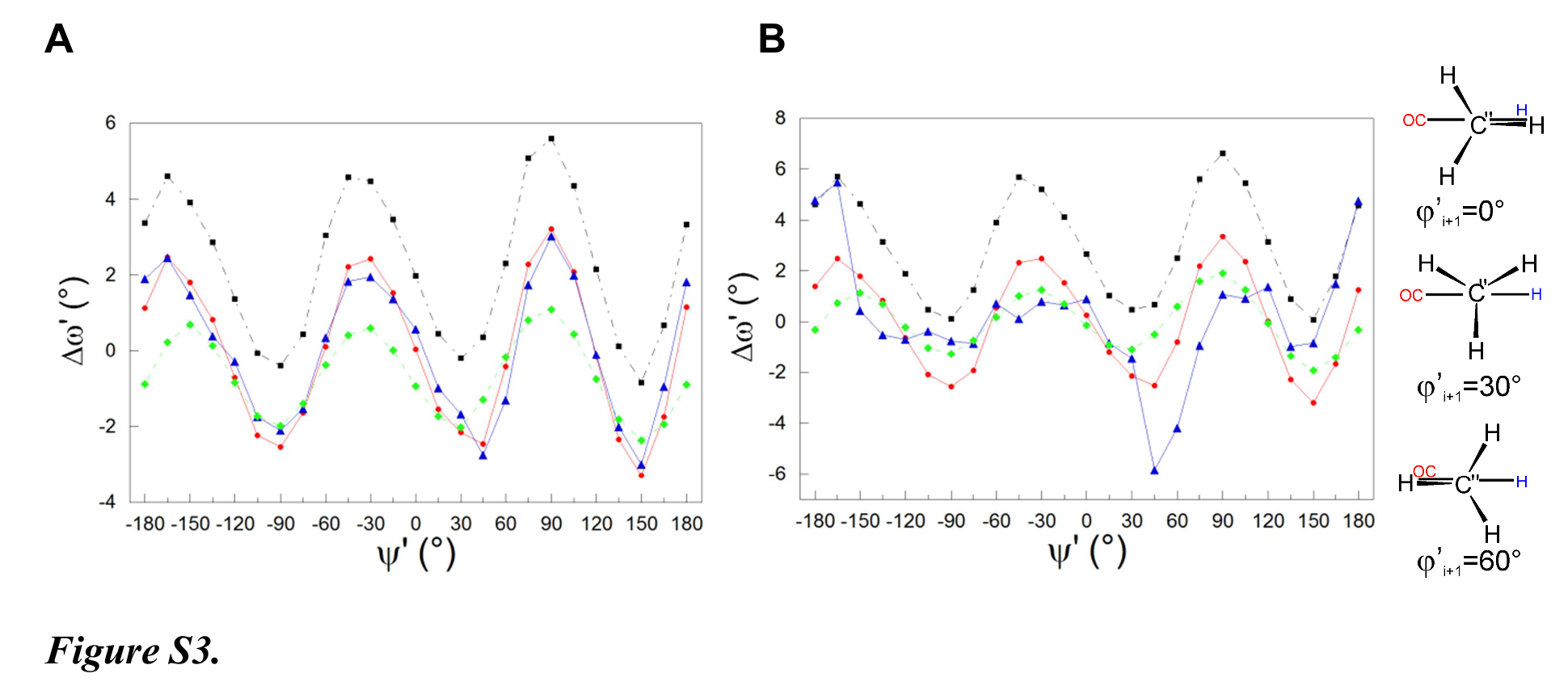

Supplement: Figure S3 — Pep model in vacuo : Δω′ variation as a function of ψ′. (A) Calculations at the PBE0/6-31G(d) level Results for the three conformers of the terminal C″ methyl group are shown (▴ϕ′i+1 = 0°; ▪ϕ′i+1 = 30°; •ϕ′i+1 = 60°) together with those for the conformer ϕ′i+1 = 30° with the OCNH dihedral angle constrained to be 180° (⧫ϕ′i+1 = 30° planar form). (B) Calculations at the MP2/6-31G(d) level. Results for the three conformers of the terminal C″ methyl group are shown (▴ϕ′i+1 = 0°; ▪ϕ′i+1 = 30°; •ϕ′i+1 = 60°) together with those for the conformer ϕ′i+1 = 0° with the OCNH dihedral angle constrained to be 180° (⧫ϕ′i+1 = 0° planar form). On the right, schematic drawings of the ϕ′i+1 conformers are shown (for a larger version, see Figure S1). (TIF) [file pone.0024533.s004.tif]

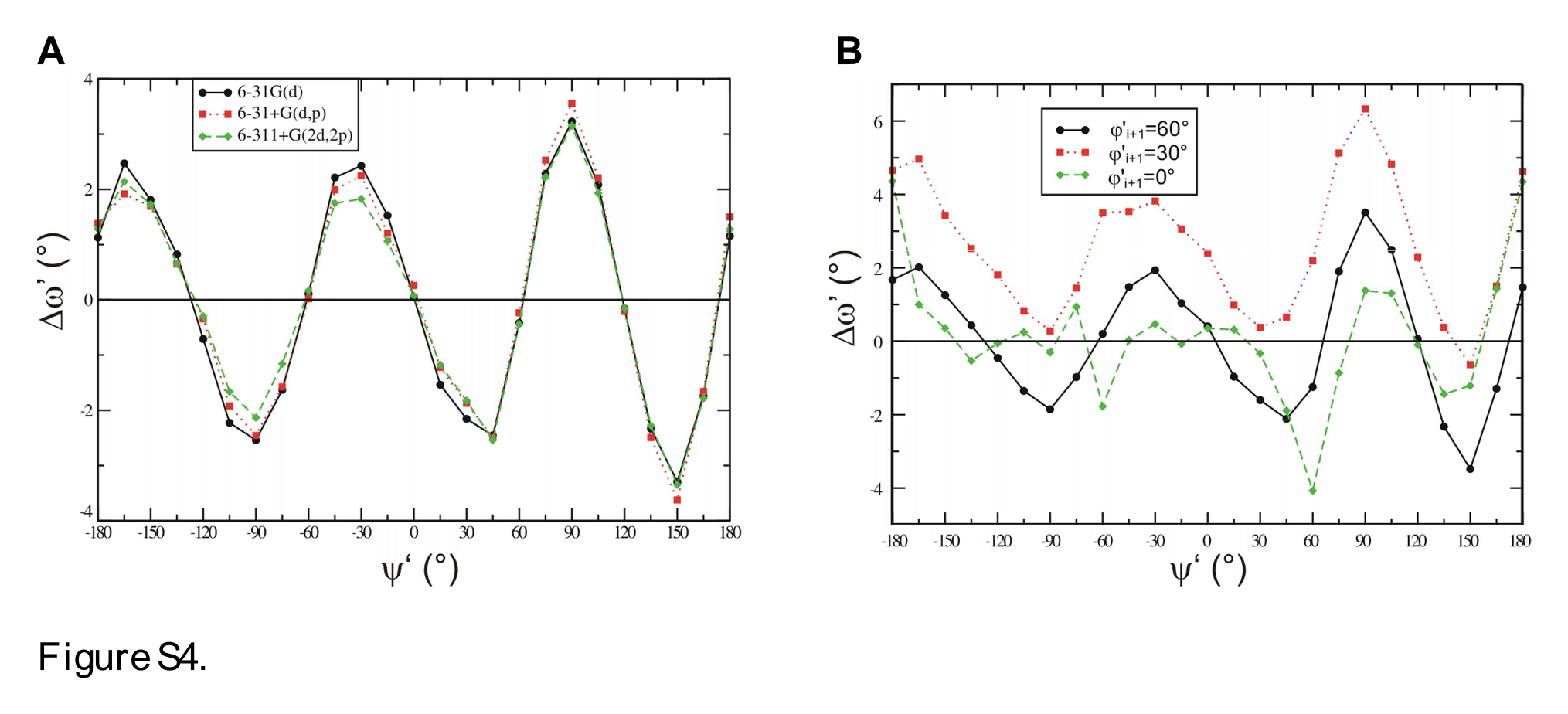

Supplement: Figure S4 — Pep model in vacuo : Δω′ variation as a function of ψ′. (A) Calculations for the conformer ϕ′i+1 = 60° at different levels of theory: •PBE0/6-31G(d), ▪PBE0/6-31+G(d,p) and ⧫PBE0/6-311+G(2d,2p) (B) Calculations at the MP2/6-31G(d,p) level for the three conformers of the terminal C″ methyl group: •ϕ′i+1 = 60°, ▪ϕ′i+1 = 30° and ⧫ϕ′i+1 = 0°. (TIF) [file pone.0024533.s005.tif]

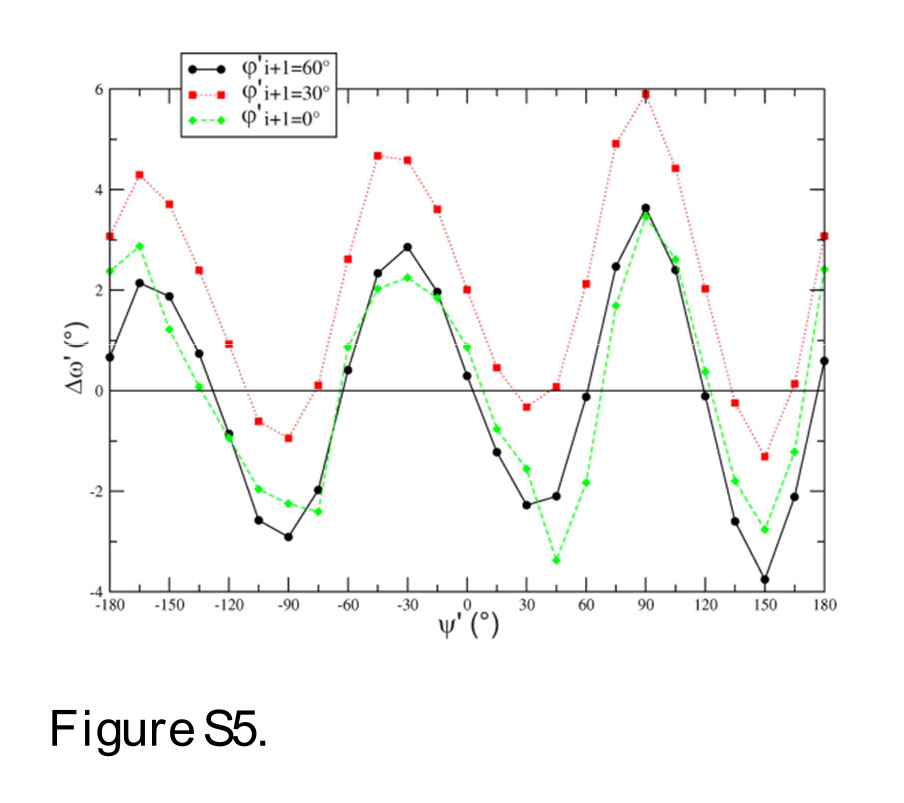

Supplement: Figure S5 — Pep model in vacuo : Δω′ variation as a function of ψ′. DFT calculations adopting the M05-2X functional. (TIF) [file pone.0024533.s006.tif]

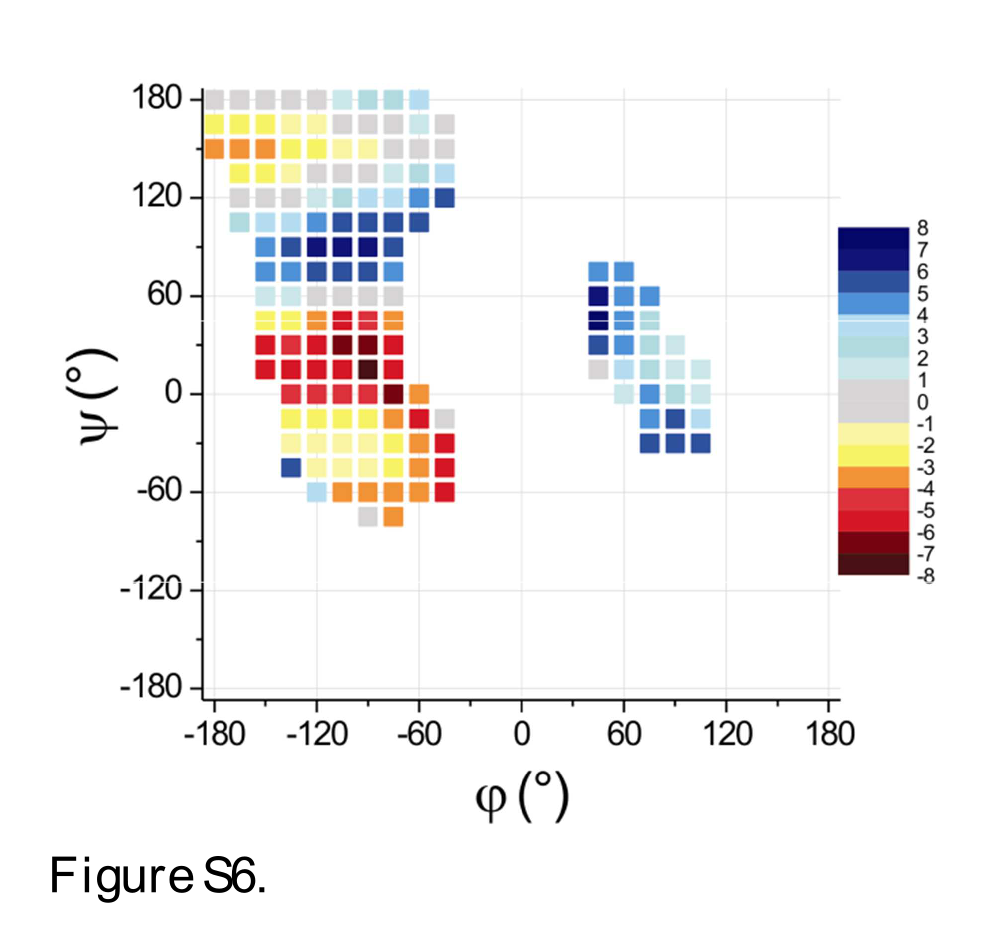

Supplement: Figure S6 — Ala1 model in vacuo . Dependence of Δω on peptide conformation. (TIF) [file pone.0024533.s007.tif]

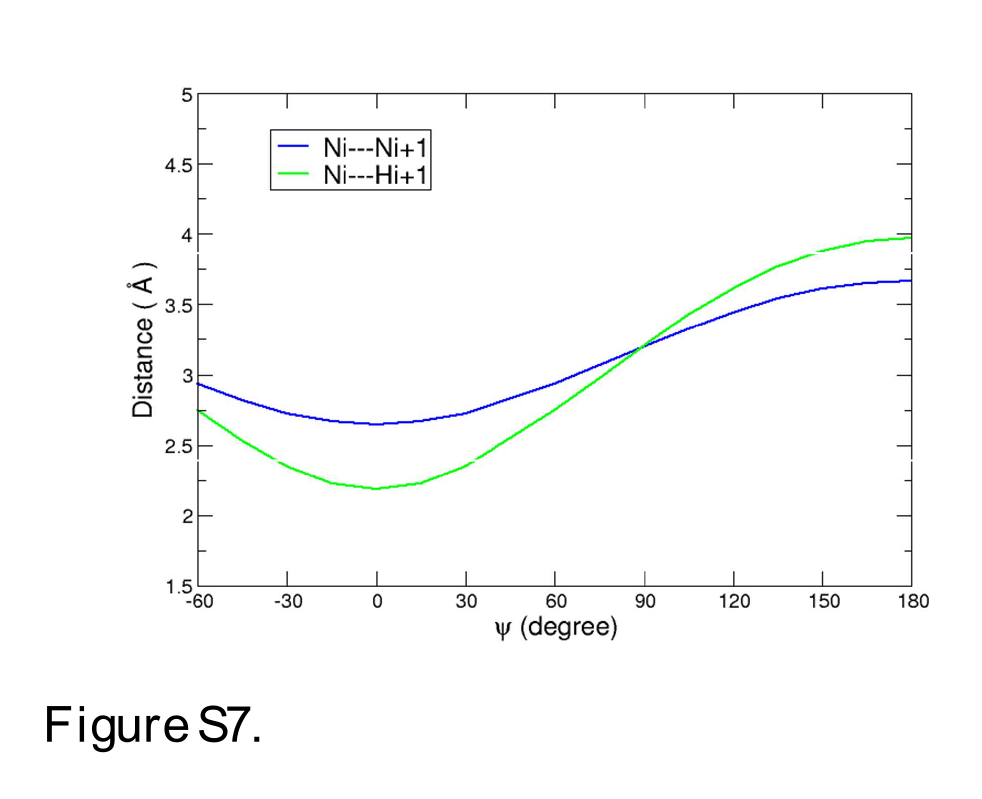

Supplement: Figure S7 — Dependence of Ni-Ni+1 (blue) and Ni-Hi+1 (green) on the ψ angle. (TIF) [file pone.0024533.s008.tif]

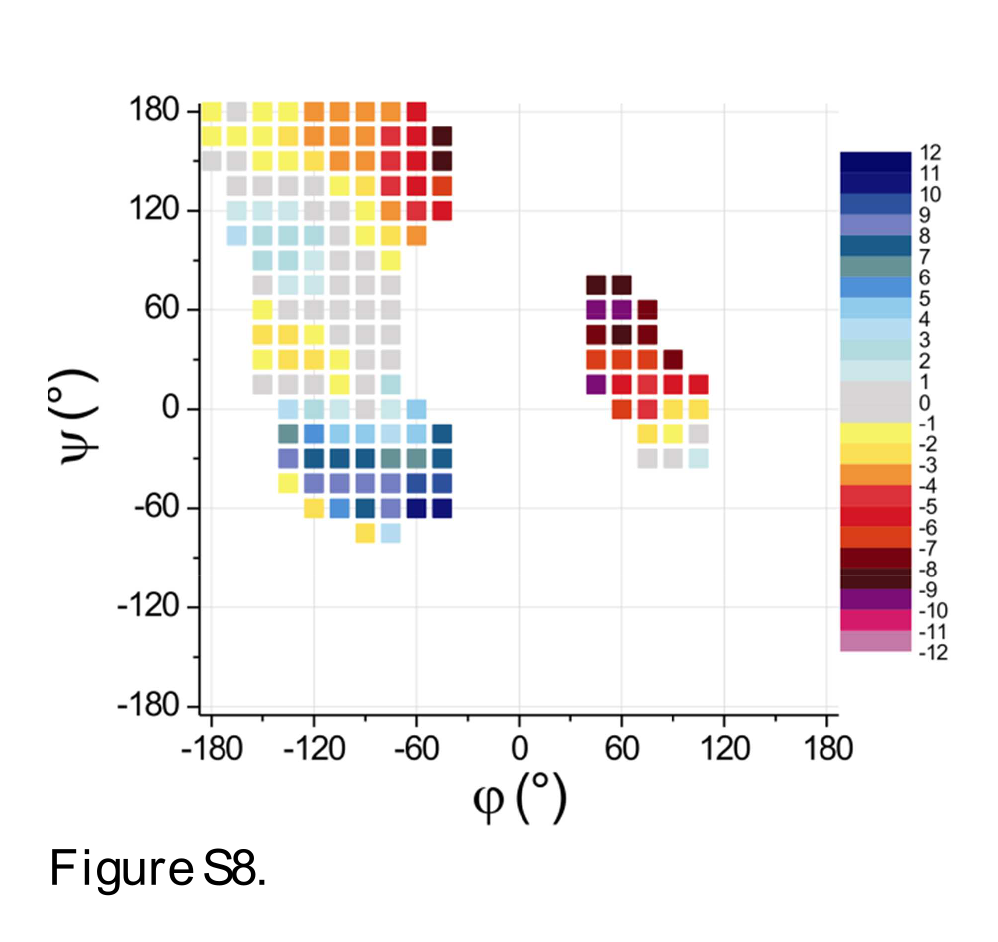

Supplement: Figure S8 — Ala1 model in vacuo . Dependence of θC on peptide conformation. (TIF) [file pone.0024533.s009.tif]

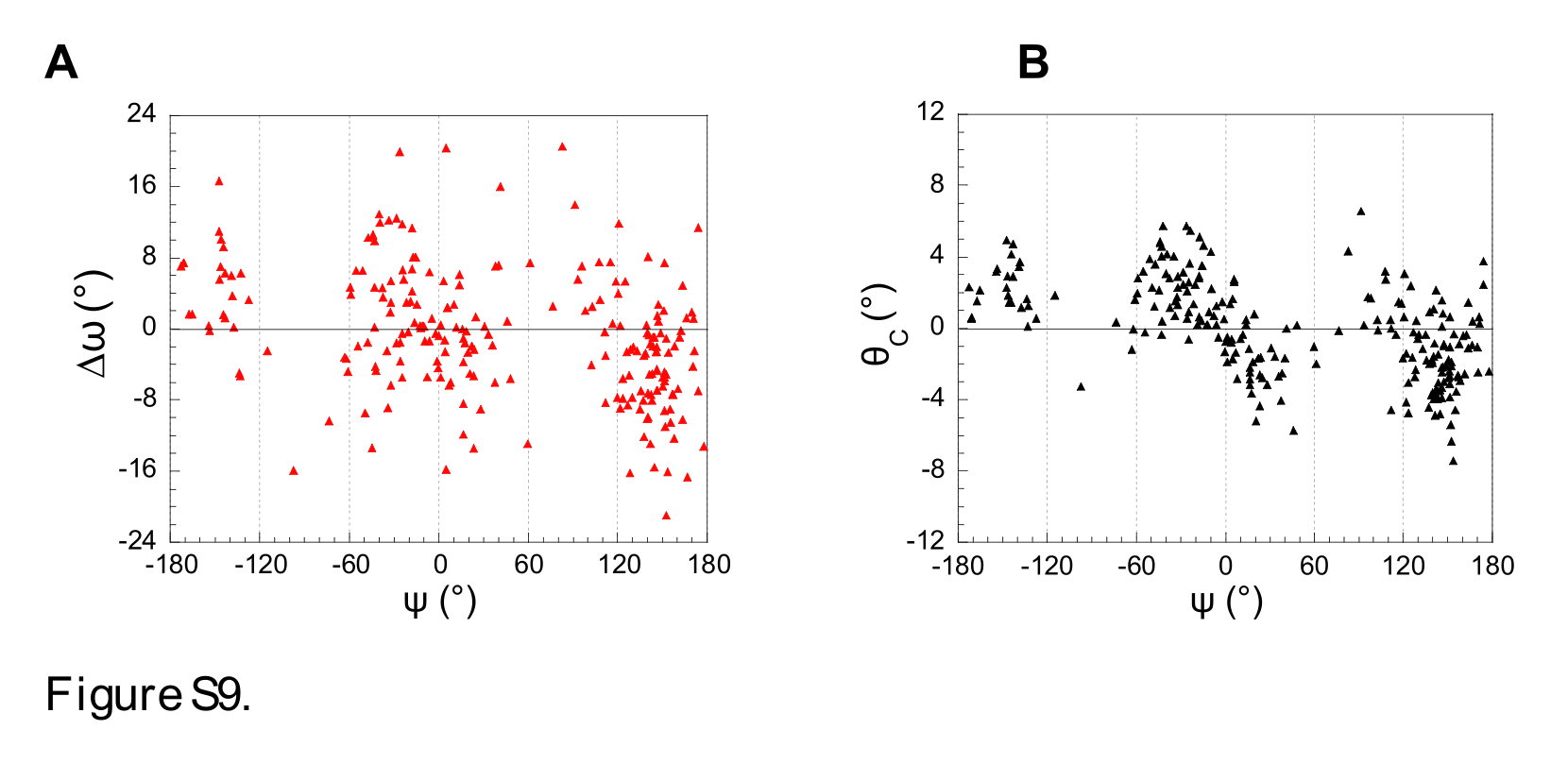

Supplement: Figure S9 — CSD small molecule structure survey. Dependence of peptide bond geometrical parameters on peptide conformation. (A) Δω variation as a function of ψ (B) θC variation as a function of ψ. (TIF) [file pone.0024533.s010.tif]

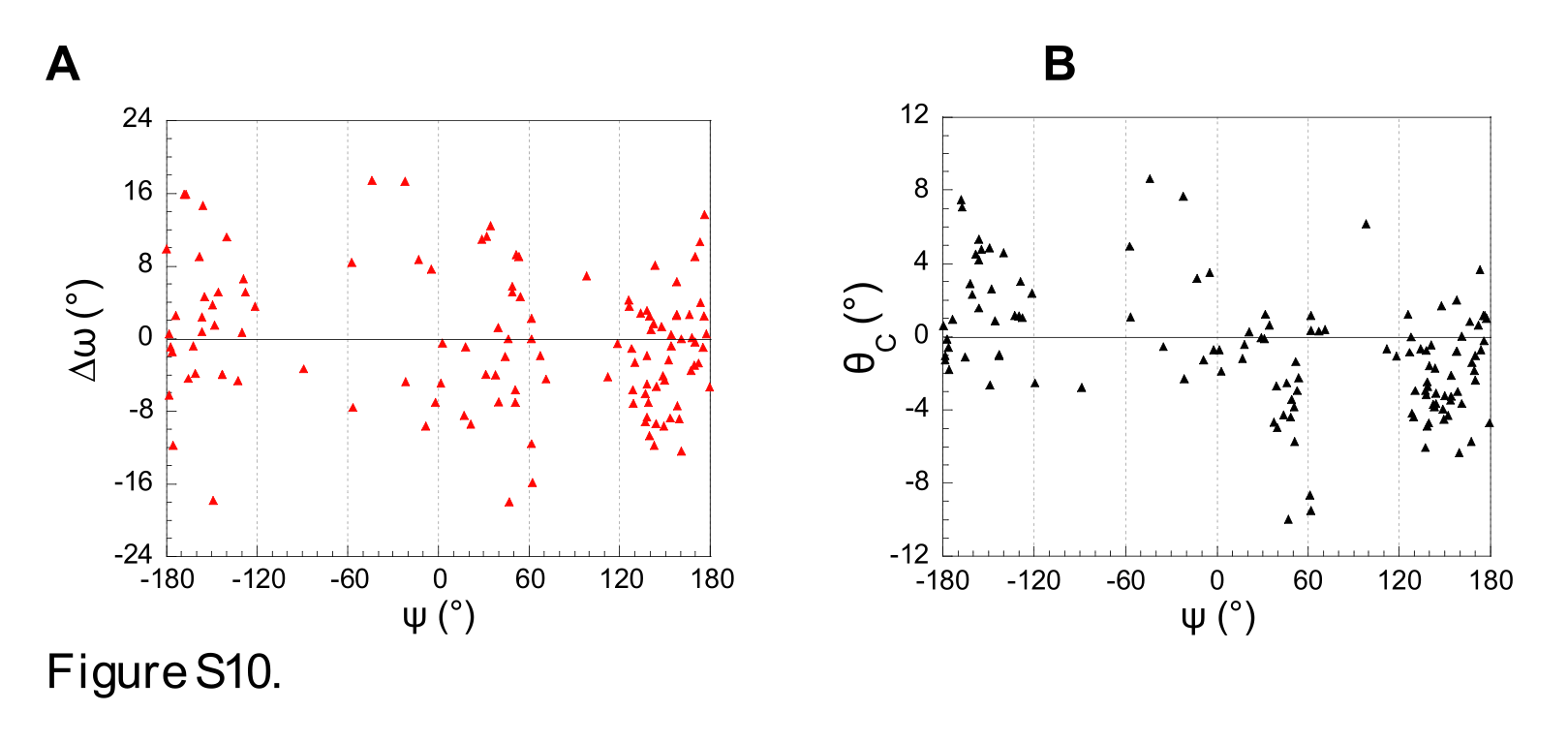

Supplement: Figure S10 — CSD small molecule structure survey of tertiary amides (see Table S1). Dependence of peptide bond geometrical parameters on peptide conformation. (A) Δω variation as a function of ψ (B) θC variation as a function of ψ. Only peptide planes in trans conformation were included in the analysis. Accurate peptide models were selected by restricting the survey to the structures determined at low temperature (T<200K) with an R-factor lower than 0.05. (TIF) [file pone.0024533.s011.tif]

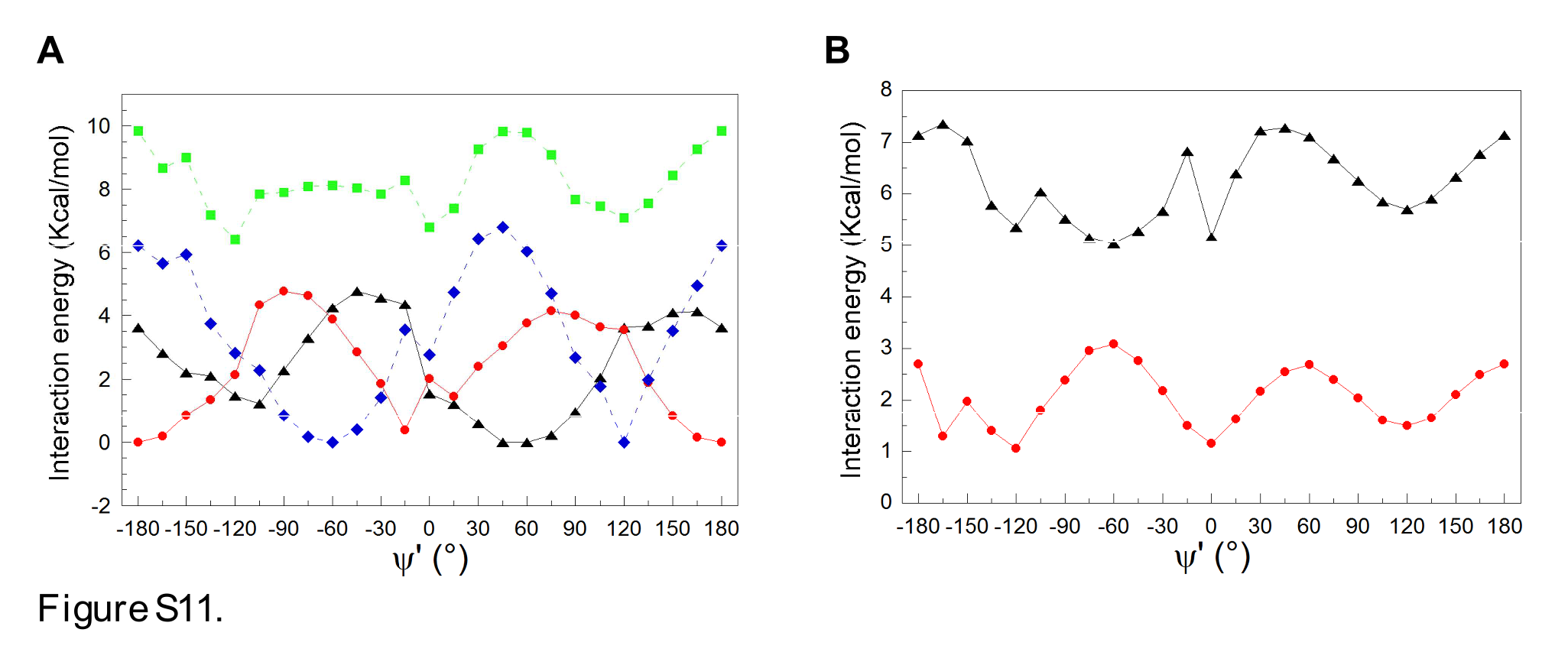

Supplement: Figure S11 — NBO analysis of Pep at the PBE0 level. (A) Orbital interaction energy between the Cα σ system and the CO π system as a function of ψ′ (▴ Cα-Cβ σ→CO π* + CO π→Cα-Cβ σ*; • Cα-S σ→CO π* + CO π→Cα-S σ*; ⧫ Cα-Hα σ→CO π* + CO π→Cα-Hα σ*; ▪ Sum of the above three contributions) (B) Orbital interaction energy for (▴) Cα-X σ→CO π* and (•) CO π→Cα-X σ* as a function of ψ′. With Cα-X we represent the sum of the three Cα-Cβ, Cα-S, and Cα-Hα contributions. It is worth noting that, due to the non-perfect separation between σ and π systems, the Cα substituent bonds interact also with the CO σ bond. These small contributions are not included in the figure, explaining why some deviations from the ideal sinusoidal behavior can be found. (TIF) [file pone.0024533.s012.tif]

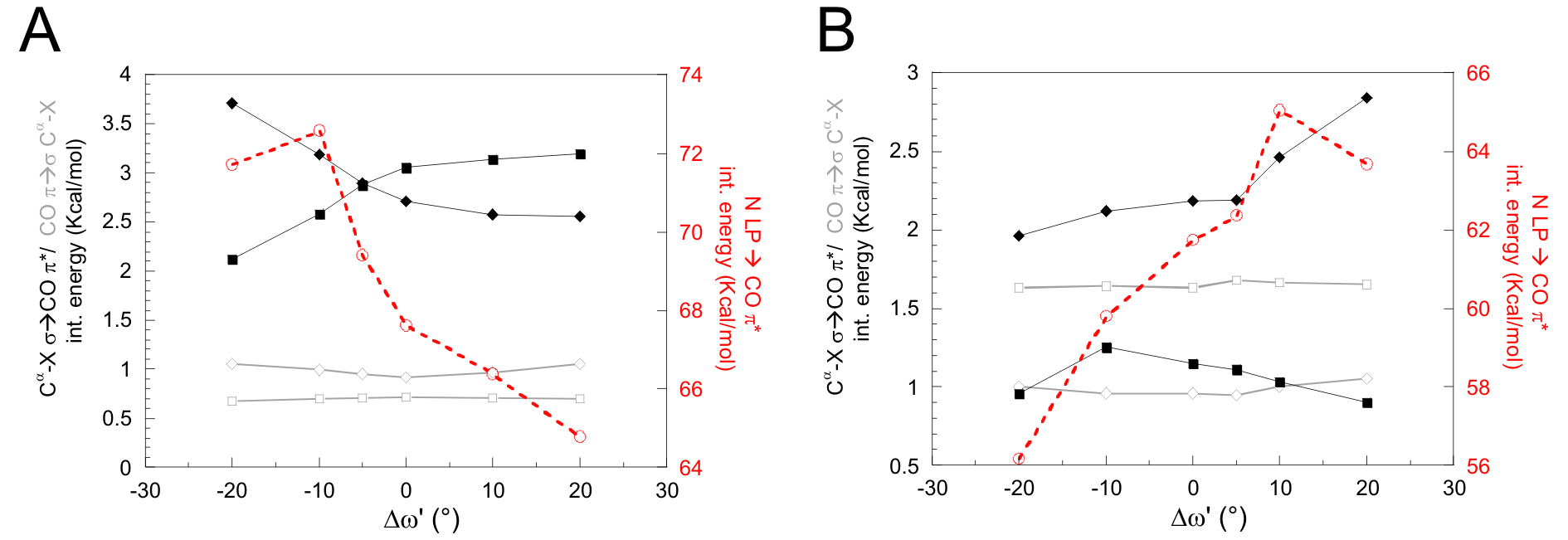

Supplement: Figure S12 — NBO analysis for different Δω′ values in two representative conformers of Ala1-Solv model. (A) Orbital interaction energy versus Δω′ for the ϕ = −135°, ψ = 150° conformer (▪ Cα-Hα σ→CO π*; □ CO π→Cα-Hα σ*;♦ Cα-Cβ σ→CO π*;◊ CO π→Cα-Cβ σ*;○ N n→CO π*); (B) Orbital interaction energy versus Δω′ for the ϕ = −60°, ψ = −45° conformer (▪ N-Cα σ→CO π*;□ CO π→N-Cα σ*;♦ Cα-Cβ σ→CO π*;◊ CO π→Cα-Cβ σ*;○ N n→CO π*). (TIF) [file pone.0024533.s013.tif]

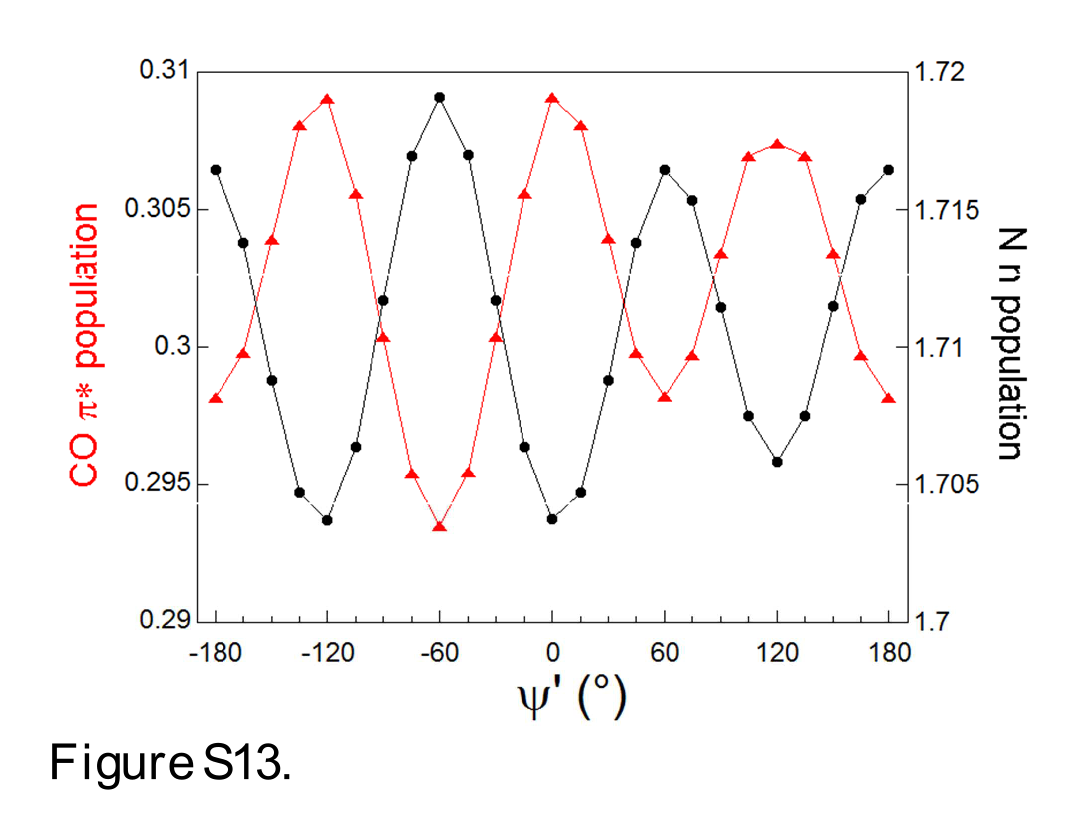

Supplement: Figure S13 — Population of the CO nonbonding π* orbital (▴) and the N n orbital (▪) as a function of ψ′ in Pep. (TIF) [file pone.0024533.s014.tif]
