# Supplementary material for: Peptide Bond Distortions from Planarity: New Insights from Quantum Mechanical Calculations and Peptide/Protein Crystal Structures
Source: PLoS One. 2011 Sep 16;6(9):e24533. doi: 10.1371/journal.pone.0024533 (PMC3174960; doi:10.1371/journal.pone.0024533)
Supplement: Table S1 — Surveys of CSD small molecule crystal structures. (DOCX) [file pone.0024533.s015.docx]

**Table S1. Surveys of CSD small molecule crystal structures**

| **Search fragment** | **Refcodes of the structures** |
| --- | --- |
| a)  C-N-CH-CO-NH-C  \|  C | AAGAGG10, ADUHEM, AFUWON, AGAQUV, AJUNEY, AQUWOY, AXUYIB, BAWGIQ, BIHYAS10, CAHWEN, CAMVES, CAMVES01, CEBBER, CEPQIY, CEPQOE, CEPQUK, CIKNOA, CIPQEX, COFXUR, COJFIR, CUJHOE, DACMAW, DAKQUB, DALPUC, DIWQUW, EVEDEO, EVEZOU, EYOCAW, EYOCEA, EZIROU, EZIRUA, FALLIO, FAQYEC, FEDFUQ, FEDGAX, FEDGAZ, FEDGEB, FEDGIF, FEDGOL, FIKFUB, FIQKAS, GADCAQ, GIXGIE, GOMKAU, GUFKEX, GUKXOZ, GUWWEA, HEJRAP, HOFNAR01, HOHLOG, HOHMAT, HOHMUN, HORKEF, IDOFOX, IGAVER, INOTIO, IWANID, JATTEE, JAYWUC, JAYYEO, JECYUL, JINDIT, JINWUZ, JUJBOF, KEBMIO, KELYIK, KELYIK01, KELYOQ, KELYOQ01, KEPDEO01, LALWIF, LENMAT, LIXPEO, LIXQAL, LIXQEP, LIXQIT, LIXQOZ, LOBQAU, LOKTIP, MAKLEQ, MIJVEH, MISSIQ, MIXRIU, MIXROA, NAZJEE, ODETOH, OGATIA, OGATOG, OGAVAU, PASYAJ, PATFAR, PATPIK, PEMDUH, PERYER, PEZNAJ, PIZYIH, PUYRAC, QAGBIK, QANQUR, QIRRUE, QOCXUB, QOHDUM, REVHOQ, REVHUW, REXYEY01, REZSOF, RIVLUE, RUZWUE, SIBXUX, SIHPEF, SILTUD, SIRVEV, SOHFAW01, TALZUC, TEGQIG, TICCEO, TICLOG, TIFKAU, TIJKIH01, TLALAN11, TUTMEA, UCIHEU, UHUSUL, VAYNEP, VISCOQ, WAHFER, WEHXAJ, WEHXEN, WIFQOS, WILSEQ, WILSIU, WILSOA, WUVTAI, XACJAM, XENLAE, XENVIW, XENVOC, XEQGEG, XIMQIU, XISVAX, XOBYOD, XOSFAM, YIHZIZ, YIHZOF, YIZXUA, ZADHOB, ZADHUH, ZADJET, ZAQVUI, ZIKKEJ01, ZZZIFQ01 |
| b)  N-C-CO-N-C  \|  C | ABOSUG, AFAFAP, AFURAV, AGAQUV, AHANEC, AJACIX, AJACOD, AXUYIB, BIHYAS10, BOGZEC, CAMVES, CAMVES01, CASKAJ, CEBBIV, CEWTOO, CIFNEL, CIPQEX, COQSUX, DEKFAB, DIWQUW, EABXEK, FAQYEC, FIQTUV, FOBFEH, GERROL, HEJRAP, HEMNAO, IDUZAI, IFAHED, IFAXET, IGACID, IQOSOW, JECVUJ, JEQKIA, JINWUZ, JOFFAM, JUJBOF, JUQYEZ, KEBMIO, LEBDEB, LIKNUO, LIYWUM, MAJUSB, MEQSAD, MIGROK, MISSIQ, MIXRIU, MIXROA, NAFXIC, NAZJEE, NEBWUM, NIGROL, NOBQAW, NODNAW, NOVCIK, PAMFEP, PAMFUF, PEMDUH, PENDOB, PENFET, PEZNAJ, QALTUT, QEXBIF, QIRRUE, QUGWIY, QUGWOE, RIVLUE, ROHDUO, ROPVUN, ROPWAU, ROPWEY, ROPWIC, ROPWOI, SILTUD, TAJPOK, TIMDUO, TORHAK, VACPUL, VAWYIC, WEHXAJ, WEHXEN, WOBLAB, XACJAM, XASREO, XASXEV, XEBSED, XEHDET, XENVIW, XIBYUD, XIVFIS, XOBYOD, XOCZUL, XOSFAM, ZEZTIH, ZEZTIH10, ZIKKEJ01, YOMXII |
